# CTCF counter-regulates cardiomyocyte development and maturation programs in the embryonic heart

**DOI:** 10.1371/journal.pgen.1006985

**Published:** 2017-08-28

**Authors:** Melisa Gomez-Velazquez, Claudio Badia-Careaga, Ana Victoria Lechuga-Vieco, Rocio Nieto-Arellano, Juan J. Tena, Isabel Rollan, Alba Alvarez, Carlos Torroja, Eva F. Caceres, Anna R. Roy, Niels Galjart, Paul Delgado-Olguin, Fatima Sanchez-Cabo, Jose Antonio Enriquez, Jose Luis Gomez-Skarmeta, Miguel Manzanares

**Affiliations:** 1 Centro Nacional de Investigaciones Cardiovasculares (CNIC), Madrid, Spain; 2 Centro de Investigación Biomédica en Red de Enfermedades Respiratorias (CIBERES), Madrid, Spain; 3 Centro Andaluz de Biología del Desarrollo (CABD), CSIC-Universidad Pablo de Olavide-Junta de Andalucía, Seville, Spain; 4 Translational Medicine, The Hospital for Sick Children, Toronto, Ontario, Canada; 5 Department of Molecular Genetics, University of Toronto, Toronto, Ontario, Canada; 6 Department of Cell Biology and Genetics, Erasmus MC, Rotterdam, The Netherlands; 7 Heart and Stroke Richard Lewar Centre of Excellence, Toronto, Ontario, Canada; Stanford University School of Medicine, UNITED STATES

## Abstract

Cardiac progenitors are specified early in development and progressively differentiate and mature into fully functional cardiomyocytes. This process is controlled by an extensively studied transcriptional program. However, the regulatory events coordinating the progression of such program from development to maturation are largely unknown. Here, we show that the genome organizer CTCF is essential for cardiogenesis and that it mediates genomic interactions to coordinate cardiomyocyte differentiation and maturation in the developing heart. Inactivation of *Ctcf* in cardiac progenitor cells and their derivatives *in vivo* during development caused severe cardiac defects and death at embryonic day 12.5. Genome wide expression analysis in *Ctcf* mutant hearts revealed that genes controlling mitochondrial function and protein production, required for cardiomyocyte maturation, were upregulated. However, mitochondria from mutant cardiomyocytes do not mature properly. In contrast, multiple development regulatory genes near predicted heart enhancers, including genes in the *IrxA* cluster, were downregulated in *Ctcf* mutants, suggesting that CTCF promotes cardiomyocyte differentiation by facilitating enhancer-promoter interactions. Accordingly, loss of CTCF disrupts gene expression and chromatin interactions as shown by chromatin conformation capture followed by deep sequencing. Furthermore, CRISPR-mediated deletion of an intergenic CTCF site within the *IrxA* cluster alters gene expression in the developing heart. Thus, CTCF mediates local regulatory interactions to coordinate transcriptional programs controlling transitions in morphology and function during heart development.

## Author summary

Properly regulated gene expression in time and space during development and differentiation requires not only transcriptional inputs, but also specific structuring of the chromatin. CTCF is a DNA binding factor that is believed to be critical for this process through binding to tens of thousands of sites across the genome. Despite the knowledge gained in recent years on the role of CTCF in genome organization, its functions *in vivo* are poorly understood. To address this issue, we studied the effect of genetically deleting CTCF in differentiating cardiomyocytes at early stages of mouse development. Surprisingly only a fraction of genes change their expression when CTCF is removed. Importantly, misregulated genes control opposing genetic programs in charge of development and patterning on one hand, and cardiomyocyte maturation on the other. This imbalance leads to faulty mitochondria and incorrect expression of cardiac patterning genes, and subsequent embryonic lethality. Our results suggest that CTCF is not necessary for maintenance of global genome structure, but coordinates dynamic genetic programs controlling phenotypic transitions in developing cells and tissues.

## Introduction

The coordinated deployment of genetic programs during lineage commitment and differentiation is a hallmark of developmental processes. Cell specification and maturation are coordinated by controlled activation and repression of specific gene expression programs. In the heart, the first functional organ in the embryo, activation of a core set of cardiogenic transcription factors controls specification of cardiac progenitor cells [1]. Shortly after, high expression of genes encoding sarcomeric components defines the contractile cardiomyocyte as early as embryonic day (E) 8.5. Cardiomyocytes then mature by further sarcomere assembly [2], and increased mitochondrial biogenesis [3, 4], and finally exit the cell cycle and become binucleated at early postnatal stages [5].

Even though the genes and regulatory networks controlling morphogenesis and function in the heart are well characterized [6], the events that coordinate the progression from differentiation to maturation are not understood. Recent studies using both mouse and human pluripotent cells have revealed that epigenomic landscapes and chromatin signatures dynamically change during cardiomyocyte differentiation [7, 8], suggesting that chromatin structure might control cardiogenesis. Chromatin conformational changes allow physical interaction of distal regulatory elements in the genome. However, the chromatin interactions controlling expression of cardiac development and maturation are poorly understood.

The study of genome function during the last decade [9, 10] has provided an initial understanding of how functional elements scattered throughout the genome act coordinately to control gene activity. The advent of tools to analyze interactions between distal regions of chromatin [11] has allowed detailed mapping of the three-dimensional genome structure [12, 13] and its organization in distinct regulatory domains [14]. However, how these domains are established, and their function in gene expression regulation are poorly understood. CTCF (CCCTC-binding factor) is one of the best described architectural proteins with a role in chromatin structure organization. Through sequence specific binding to DNA, CTCF acts as a barrier for heterochromatin spreading, establishes boundaries between adjacent topologically associating domains (TADs), defines insulator elements that block enhancer activity on promoters, and contributes to enhancer-promoter interactions [15, 16]. Loss of function studies using knock-out and knock-down approaches have shown that CTCF is essential in early embryo development [17–19]. Conditional deletion of *Ctcf* in different developmental contexts leads to defects in cell cycle progression [17], increased apoptosis [20, 21], and its deletion in postmitotic neurons leads to decreases in the expression of clustered protocadherin genes [22]. Yet, we still do not fully understand how CTCF controls chromatin structure to coordinate gene expression.

Here, we have studied how CTCF regulates gene expression in the context of the developing mammalian heart. We deleted *Ctcf* in a population of cardiac progenitor cells, which results in cardiac malformations and embryonic death. Analysis of global transcriptional changes preceding morphological defects caused by loss of CTCF showed downregulation of the cardiac developmental program, and concomitant upregulation of programs involved in cardiomyocyte maturation. This suggests that *Ctcf* deletion causes a premature arrest of cardiac development and concomitantly promotes cardiomyocyte maturation. Thus, CTCF mediates local chromatin interactions to coordinate transcriptional programs that control developmental transitions in the heart.

## Results

### Cardiac specific deletion of *Ctcf* leads to embryonic lethality

**Fig 1:**
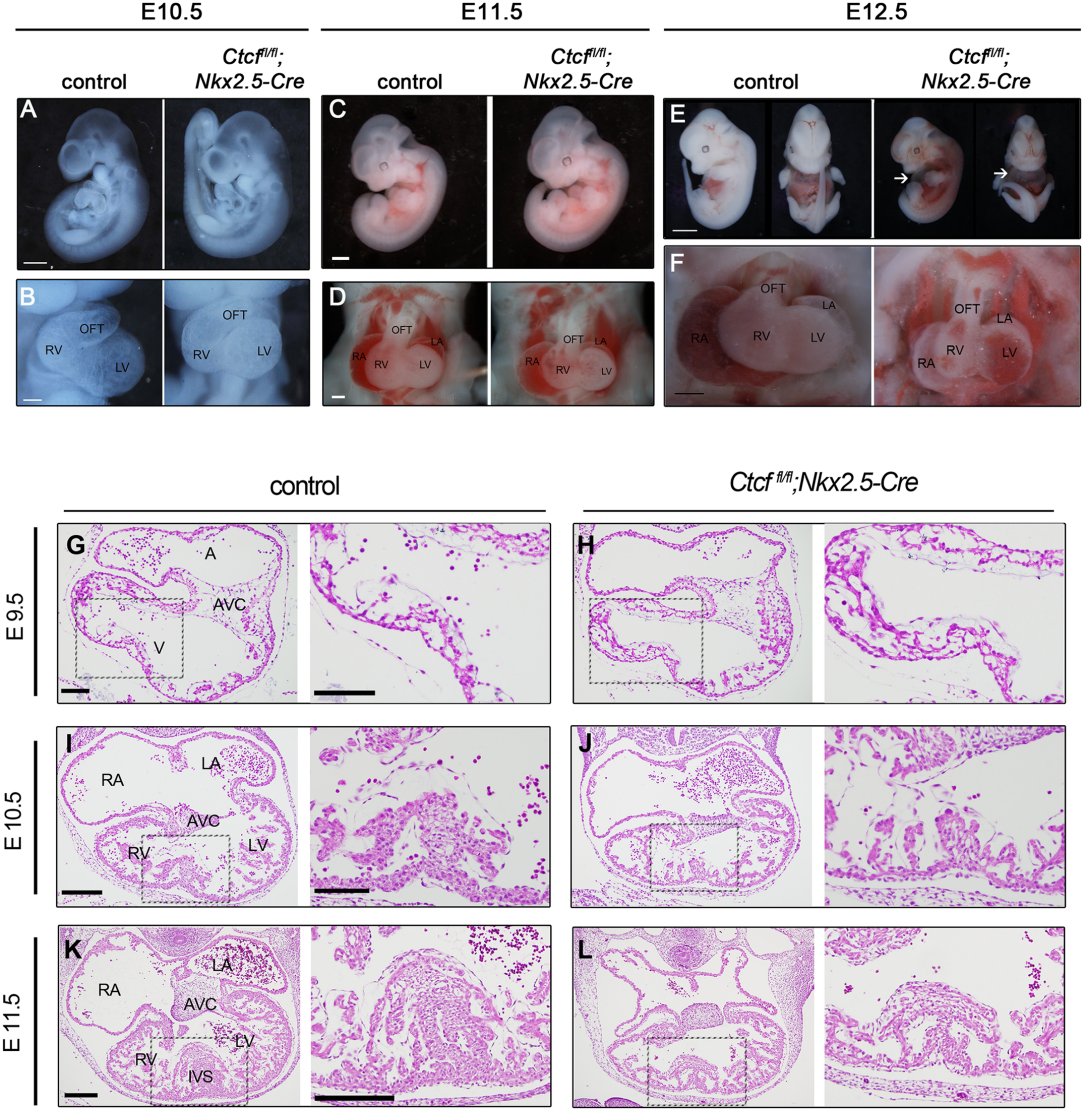
Morphological defects in *Ctcf* mutant embryonic hearts. Whole mount control and mutant (*Ctcf*^*fl*/*fl*^;*Nkx2-5-Cre*) embryos at E10.5 (A, B), E11.5 (C, D) and E12.5 (E, F). Arrows in (E) point to the pericardial edema present in mutant embryos. Higher magnification of the heart show morphological defects in *Ctcf* mutant hearts becoming manifest at E12.5 (F). (G-L) Sections from control and mutant embryos stained with hematoxylin and eosin; higher magnifications (black dashed boxes) for each are shown on the right of each section. Disorganization of the interventricular septum and thinning of the myocardial wall is apparent in *Ctcf* mutant hearts at E11.5. A, atria; V, ventricle; RA, right atria; LA, left atria; RV, right ventricle; LV, left ventricle; AVC, atrioventricular canal; IVS, interventricular septum. Scale bars, 750 μm (A), 200 μm (B, D), 1 mm (C), 2 mm (E), 800 μm (F), 100 μm (G, H), 200 μm (I-L), and 100 μm in all higher magnifications.

To address the role of CTCF during development, we deleted *Ctcf* in cardiac progenitor cells and their derivatives by using a floxed allele [17] and the *Nkx2*.*5*-*Cre* driver, which starts acting as early as E7.5-E8.0 in cardiomyocyte precursors in the cardiac crescent [23]. E10.5 or E11.5 mutant (*Ctcf*^*fl/fl*^;*Nkx2*.*5-Cre*) embryos appeared normal and showed no gross morphological alterations in the heart (Fig 1A–1D). At E12.5, mutant embryos presented pericardial edema (Fig 1E) and the cardiac chambers did not expand properly (Fig 1F). Histological examination showed no defects in E9.5 mutant hearts as compared to controls (Fig 1G and 1H). In E10.5 mutants, the four chambers and the atrioventricular canal formed properly, although the interventricular septum appeared slightly disorganized (Fig 1I and 1J). This defect was exacerbated by E11.5, when thinning of the myocardial wall was also evident (Fig 1K and 1L). No mutant embryos were recovered beyond E12.5 (S1 Table). Control *Nkx2*.*5-Cre* and compound *Ctcf*^*fl/+*^;*Nkx2*.*5-Cre* heterozygotes showed normal morphology at these stages (S1 Fig).

To understand the effect of *Ctcf* deletion in the developing heart, we determined the time point when CTCF protein was lost in cardiomyocytes. We performed co-immunostaining for CTCF and the cardiomyocyte marker TNNT2 at different stages of heart development (S2 Fig). In E9.5 mutant hearts, 46% of cardiomyocytes still had detectable nuclear CTCF, albeit at lower levels than in controls, in which all nuclei were double positive (S2A and S2B Fig; S2 Table). However, by E10.5 we were not able to identify any cardiomyocyte expressing CTCF in mutant hearts, although expression was present in endocardium (S2C and S2D Fig). The same pattern was observed at E11.5 (S2E and S2F Fig). Persistent CTCF protein in cardiomyocytes at E9.5, despite that *Nkx2*.*5*-*Cre* is active from E7.5-E8.0 [23], could be explained by protein long half-life [24, 25]. It has been recently shown that 15% of CTCF is sufficient for proper function [26]. Therefore, the remaining protein we observe can explain why morphological defects are not detected until E10.5.

We assessed whether cardiac defects were due to decreased cell proliferation or increased apoptosis. Numbers of cardiomyocytes positive for TUNEL staining (S3A–S3F, S3M and S3O Fig; S3 Table) or phosphorylated histone H3 (S3G–S3L, S3N and S3P Fig; S3 Table) were not altered in mutant hearts at E10.5 and E11.5, indicating that apoptosis and proliferation were unaffected. Accordingly, we did not observe a difference in the number of cardiomyocytes between mutants and controls at these stages (S3 Table). Our results differ from others showing that CTCF loss in other developmental systems cause increased apoptosis [20], suggesting context-specific effects of CTCF. Our results indicate that *Ctcf* is required for cardiac morphogenesis.

### Transcriptional changes in the developing heart caused by *Ctcf* deletion

To understand the function of CTCF as a transcriptional regulator in the developing heart, we analyzed the global effects of CTCF loss on gene transcription. We performed RNA-seq analysis on hearts homozygous for cardiac-specific deletion of *Ctcf* (*Ctcf*^*fl/fl*^;*Nkx2*.*5-Cre*); heterozygotes, with deletion of only one allele (*Ctcf*^*fl/+*^;*Nkx2*.*5-Cre*); and control heterozygotes for the floxed allele but not carrying the *Nkx2*.*5-Cre* driver (*Ctcf*^*fl/+*^). To identify the earliest transcriptional effects of *Ctcf* deletion this analysis was performed at E10.5, when we first observed a complete loss of CTCF in the mutants. Comparison between homozygous *Ctcf*-deleted hearts and heterozygotes or controls yielded approximately 2,000 differentially expressed genes in each case, of which roughly half were upregulated and half downregulated (S4 Table). Interestingly, comparison between heterozygotes and controls returned only 24 differentially expressed genes, including *Ctcf* itself and11 pseudogenes (S4 Table). This suggests that, at least in the developing heart [27], one functional *Ctcf* allele is sufficient for correct regulation of gene expression.

**Fig 2:**
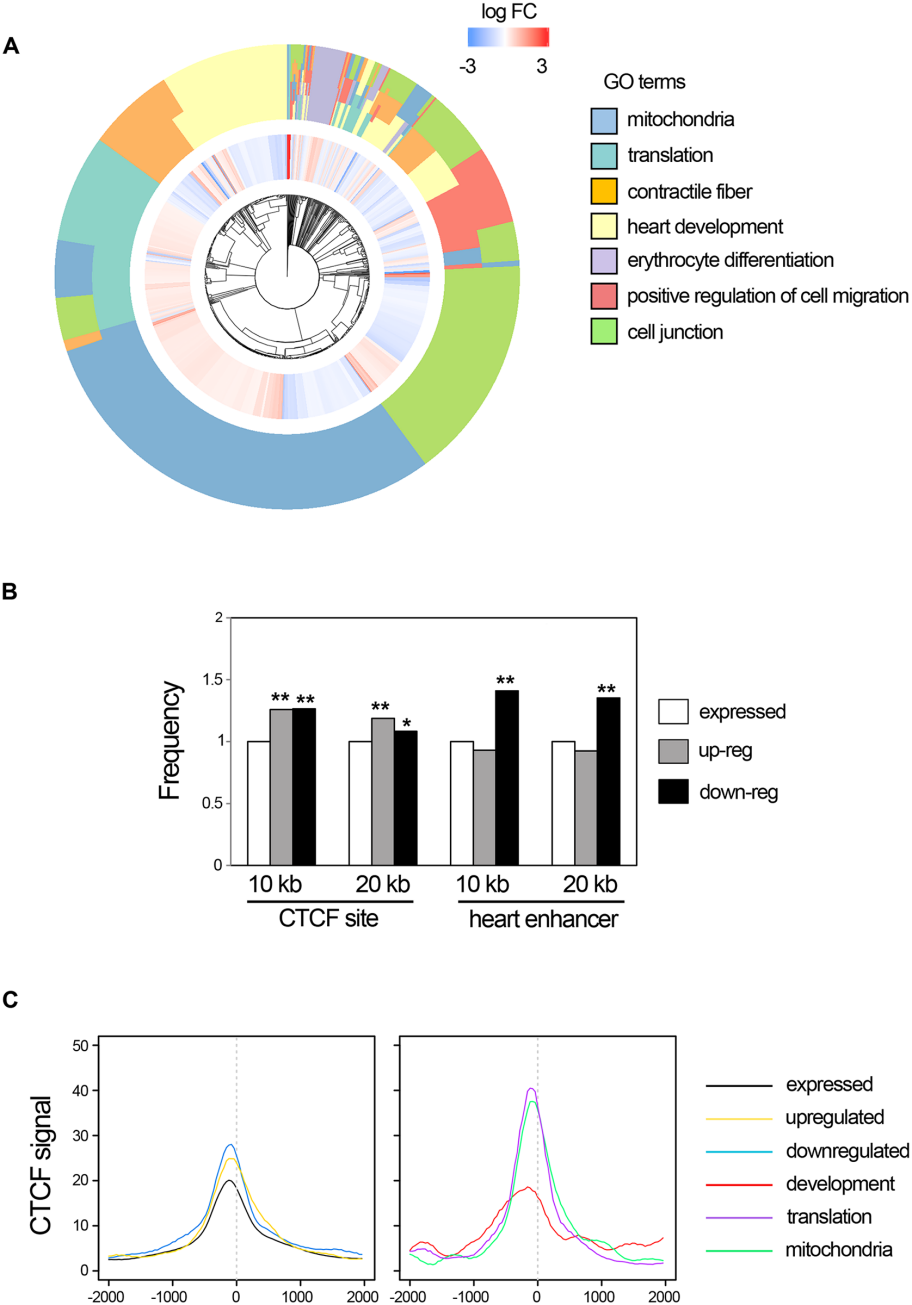
Transcriptional changes in *Ctcf* mutant hearts. (A) Iris plot showing enriched GO terms in differentially expressed genes between control and mutant E10.5 hearts (outside circle), together with changes in expression of individual genes (inner circle; red, upregulated in mutants; blue, downregulated). (B) Genes that are up- or downregulated in *Ctcf* mutant hearts are more likely to have a CTCF binding site in their vicinity (10 or 20 kb surrounding the transcriptional start site) than genes that are expressed in the embryonic heart but do not change. However, only downregulated genes are located closer to heart-specific enhancers. Frequency is expressed relative to that of expressed but unchanged genes. *, p<0.01; **, p<0.0005; Mann-Whitney test. (C) Mean-plots of the distribution of CTCF binding, as determined by ChIP-seq [10], relative to the TSS (0 on the x-axis) of different groups of genes (legend on the right). Note that the peak of the distributions is located slightly upstream of the TSS, indicative of binding to proximal promoter sequences.

Gene-ontology analysis [28] of all genes differentially expressed upon *Ctcf* deletion showed enrichment in terms related to developmental processes, including heart development, contractile fibers, translation and mitochondria (Fig 2A; S5 and S6 Tables). When we analyzed upregulated and downregulated genes separately, we found a clear distinction in the functional categories enriched in each case. Numerous upregulated genes were enriched in categories related to translation and mitochondrial function. In contrast, the downregulated genes were over represented in categories related to heart development and the sarcomere (S5 and S6 Tables).

Detailed analysis revealed that more than 300 genes related to mitochondrial function were up- and down regulated in *Ctcf* mutant hearts. Many of such genes encode subunits of mitochondrial respiratory complexes I, III, IV and V/ATP synthase, *Sdhd* in Complex II, and the large and small mitochondrial ribosome subunits (S4A Fig; S6 Table). This could suggest that CTCF controls expression of core transcriptional regulators of the mitochondrial gene program. However, none of these factors, such as *PGC-1α*, *ERRs*, or *NRF-1/2* [29], where dysregulated in mutant hearts (S4 Table). Other upregulated genes are involved in translation and encode most cytoplasmic ribosomal proteins, various initiation and elongation factors, and members of the spliceosomal complex (S4B Fig; S6 Table).

### Genomic features of genes dysregulated in the CTCF-deficient heart

CTCF organizes chromatin structure and contributes to the establishment of regulatory domains in the genome [30]. We found that genes misregulated in *Ctcf* mutant hearts do not cluster in specific genomic regions (S5A Fig), suggesting that CTCF does not control gene expression in large genomic regulatory domains similarly to what has been recently shown in embryonic stem cells [26].

We next examined CTCF chromatin binding near genes whose expression changed upon *Ctcf* deletion in the heart by mapping the distance between the transcriptional start site (TSS) and the nearest CTCF ChIP-seq peak obtained from published datasets on adult 8 week hearts [10]. We found that the up- and downregulated genes are closer to a CTCF binding site than genes whose expression does not change upon *Ctcf* deletion (S5B Fig). Arbitrarily analyzing 10 or 20 kb windows based on the above distribution, we found that TSS of down- and up-regulated genes are surrounded by CTCF binding sites more frequently than genes whose expression did not change in mutants (Fig 2B). These CTCF binding sites are conserved across multiple tissues, and we did not observe enrichment for heart-specific CTCF peaks near genes deregulated in the mutant. These results suggest that CTCF regulates gene expression mainly by binding nearby genomic regions, possibly by mediating local chromatin interactions.

CTCF could define gene regulatory domains by shielding genes from the influence of nearby enhancers or by facilitating enhancer-promoter interactions [15]. In the first scenario, *Ctcf* loss would lead to upregulation of gene expression and to downregulation in the second. To distinguish between these two possibilities, we determined the distance between dysregulated genes and the nearest heart enhancer [10]. We found that downregulated, but not upregulated, genes are significantly closer to a heart enhancer than expressed genes with no change in *Ctcf* mutants (S5B Fig). Again, this pattern is preserved when analysis is restricted to a 10 or 20 kb window surrounding the TSS of differentially expressed genes (Fig 2B). As downregulated genes are enriched in developmental regulators, these results suggest that CTCF promotes enhancer-promoter interactions in genes controlling cardiac progenitor establishment and differentiation.

The previous results suggest specific genomic features of up- or downregulated genes in *Ctcf* mutant hearts. To further explore these features, we analyzed in more detail the distribution of CTCF binding peaks on the same dataset used above surrounding the TSS of dysregulated genes. Up- and downregulated genes showed enrichment in CTCF binding immediately upstream of the TSS, and the binding signal was higher than that in genes whose expression did not change in mutants (Fig 2C), which agrees with the previous result (Fig 2B). However, when we analyzed only the dysregulated genes belonging to development, or mitochondria and translation categories (S6 Table) we observed a clear difference. Mitochondrial and translation genes showed increased CTCF binding near the TSS, but in developmental genes CTCF binding spread over more distal regions (Fig 2C). We searched promoter-proximal and distal sites for *de novo* and known sequence motifs to address the possibility that the presence of CTCF sites or other motifs would underlie the differences in distribution between categories. However, we only identified binding motifs for CTCF itself (S7 Table).

Our results suggests that *Ctcf* binds near the TSS to repress genes acting in mitochondria and regulating translation, both crucial for cardiomyocyte maturation [29]. In contrast, CTCF binding to genomic regions more distal to TSS promotes expression of genes located near heart enhancers and controlling cardiac development.

### Mitochondria do not mature properly in *Ctcf* mutant cardiomyocytes

**Fig 3:**
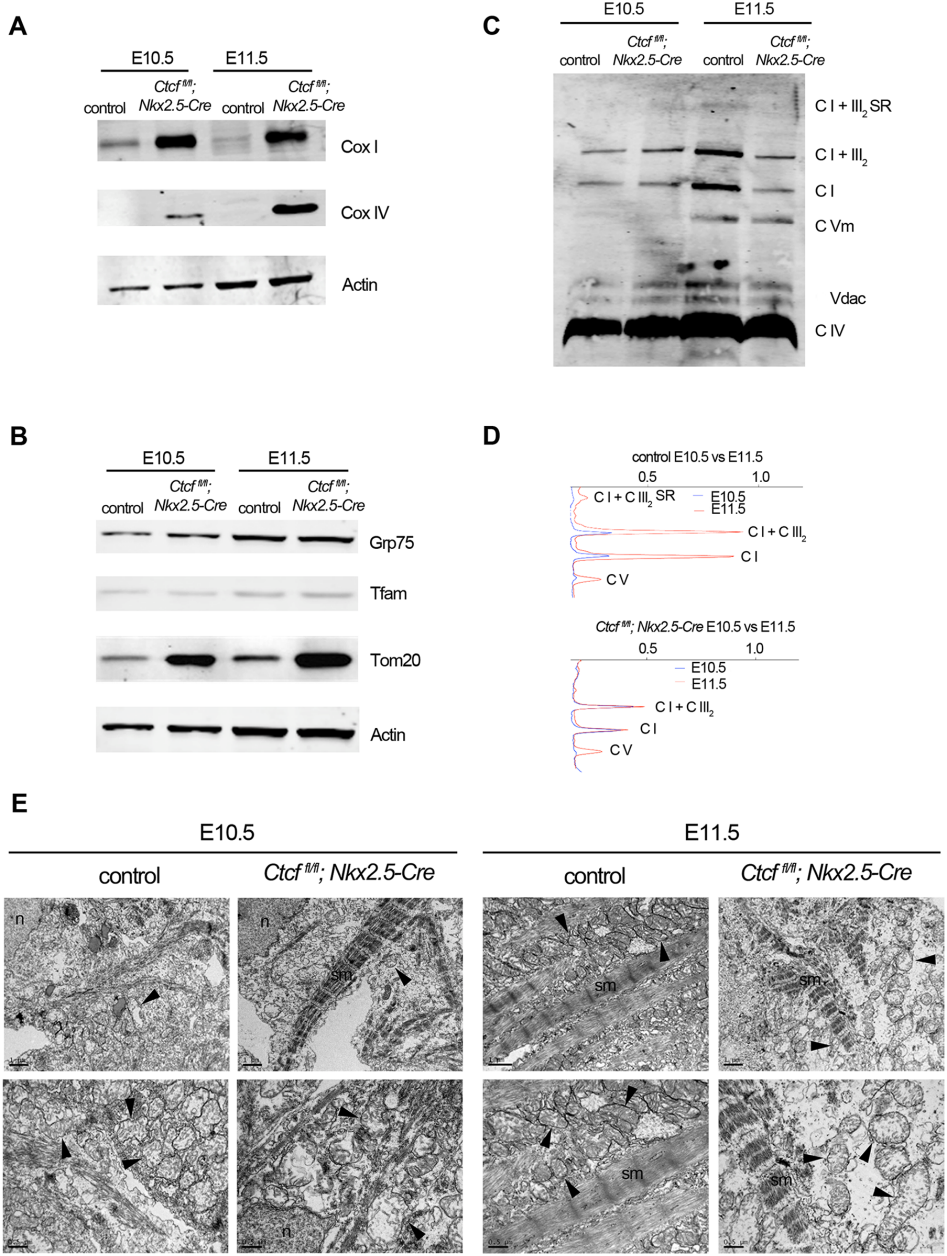
Defective mitochondrial biogenesis upon CTCF loss. Western blot of Cox I, Cox IV (A), Grp75, Tfam and Tom20 (B) of control and mutant (*Ctcf*^*fl*/*fl*^;*Nkx2-5-Cre*) hearts at E10.5 and E11.5. (C) Blue native gel of control and mutant hearts at E10.5 and E11.5 showing abundance and distribution of mitochondrial respiratory complexes (CI, CIV, CVm) and supercomplexes (CI+CIII). (D) Densitometry of blue native gel quantifying changes between E10.5 and E11.5 in complexes and supercomplexes, in control and mutant hearts. (E) Transmission Electron Microscopy at E10.5 and E11.5 in control and mutant cardiomyocytes. n, nucleus; sm, sarcomere. Arrowheads point to mitochondria. Scale bar, 1 μm top row, 0.5 μm bottom row.

Dramatic changes to the nuclear-encoded mitochondrial transcriptome, particularly of proteins involved in oxidative phosphorylation (OXPHOS) system, in *Ctcf* mutant hearts prompted us to analyze the components of this pathway in mutant and control embryonic cardiomyocytes. In agreement with the RNA-seq (S4 Table), Western blot revealed increase in Complex IV subunit I (Cox I, encoded in mitochondrial DNA) and Complex IV subunit IV (Cox IV, encoded by *Cox4i1*) in *Ctcf* mutant hearts at E10.5 and E11.5, as compared to controls (Fig 3A). Similarly increased was Tom20 (encoded by *Tomm20*) (Fig 3B), which is the mayor receptor of the mitochondrial outer membrane translocase. The abundance of other mitochondrial proteins encoded by genes whose expression did not change in *Ctcf* mutants (S4 Table), such as Grp75, a key stress chaperone regulating mitochondrial protein translocation, folding and functions [31]; and Tfam, the transcriptional regulator of mitochondrial DNA [32], was comparable between control and mutant hearts (Fig 3B).

We asked whether upregulation of OXPHOS components favors functional assembly of respiratory complexes and supercomplexes on the mitochondrial inner membrane. Blue-native gel electrophoresis revealed assembled complexes I, IV and V, and supercomplex I+III_2_ in both control and mutant hearts at E10.5 and E11.5 (Fig 3C). The mitochondrial voltage-dependent anion channel (Vdac), whose assembly is independent of respiratory complexes and supercomplexes, and whose encoding gene (*Vdac1-3*) levels did not change in mutants (S4 Table), was used as loading control. In control hearts, Complex I, both in the free form I or in the form of I+III_2_, and Complex V substantially increased from E10.5 to E11.5 (Fig 3C and 3D), consistent with maturation of mitochondrial OXPHOS [4]. In contrast, levels of I+III_2_ and I did not increase from E10.5 to E11.5 in *Ctcf* mutant hearts. Complex V was similarly increased between E10.5 and E11.5 in control and *Ctcf* mutant hearts (Fig 3C and 3D). This agrees with maturation/stabilization of the electron transport chain components being regulated independently of their production [33]. Our results suggest that despite increased transcription of subunits of CI and CIII that may lead to more complexes and supercomplexes assembled at the mitochondrial inner membrane, maturation of the respiratory chain is blunted in the *Ctcf* mutant heart.

Transmission electron microscopy (TEM) analysis revealed immature, but overall normal mitochondria with healthy cristae packaging in *Ctcf* mutant cardiomyocytes at E10.5 (Fig 3E). Mitochondria in control E11.5 cardiomyocytes have a more electro-dense matrix containing more cristae than E10.5, and are embedded in newly assembled sarcomere. This ultrastructure change agrees with blue native gel electrophoresis data and is consistent with cardiomyocyte maturation occurring between E10.5 and E11.5 [3, 4]. Mitochondria in *Ctcf* mutant cardiomyocytes at E11.5 are swollen and larger than controls, and are disorganized and scattered through the cytoplasm (Fig 3E; S6A Fig). Immunohistochemistry, targeting the mitochondrial outer membrane component Tom20, revealed disorganized mitochondria in *Ctcf* mutant cardiomyocytes at E10.5 (S6B Fig).

TEM analysis also revealed that E10.5 mutant, but not control, cardiomyocytes have long and continuous sarcomeres, which were also visible at E11.5 (Fig 3E). Quantification on images of cardiomyocytes from E10.5 hearts immunostained for α-actinin revealed comparable numbers of sarcomeric Z-bands between control and *Ctcf* mutants (S6C and S6D Fig). These results suggest that sarcomere assembly, but not sarcomeric component synthesis, is premature in the *Ctcf* mutant embryonic heart.

### Loss of CTCF disrupts the cardiac developmental program

**Fig 4:**
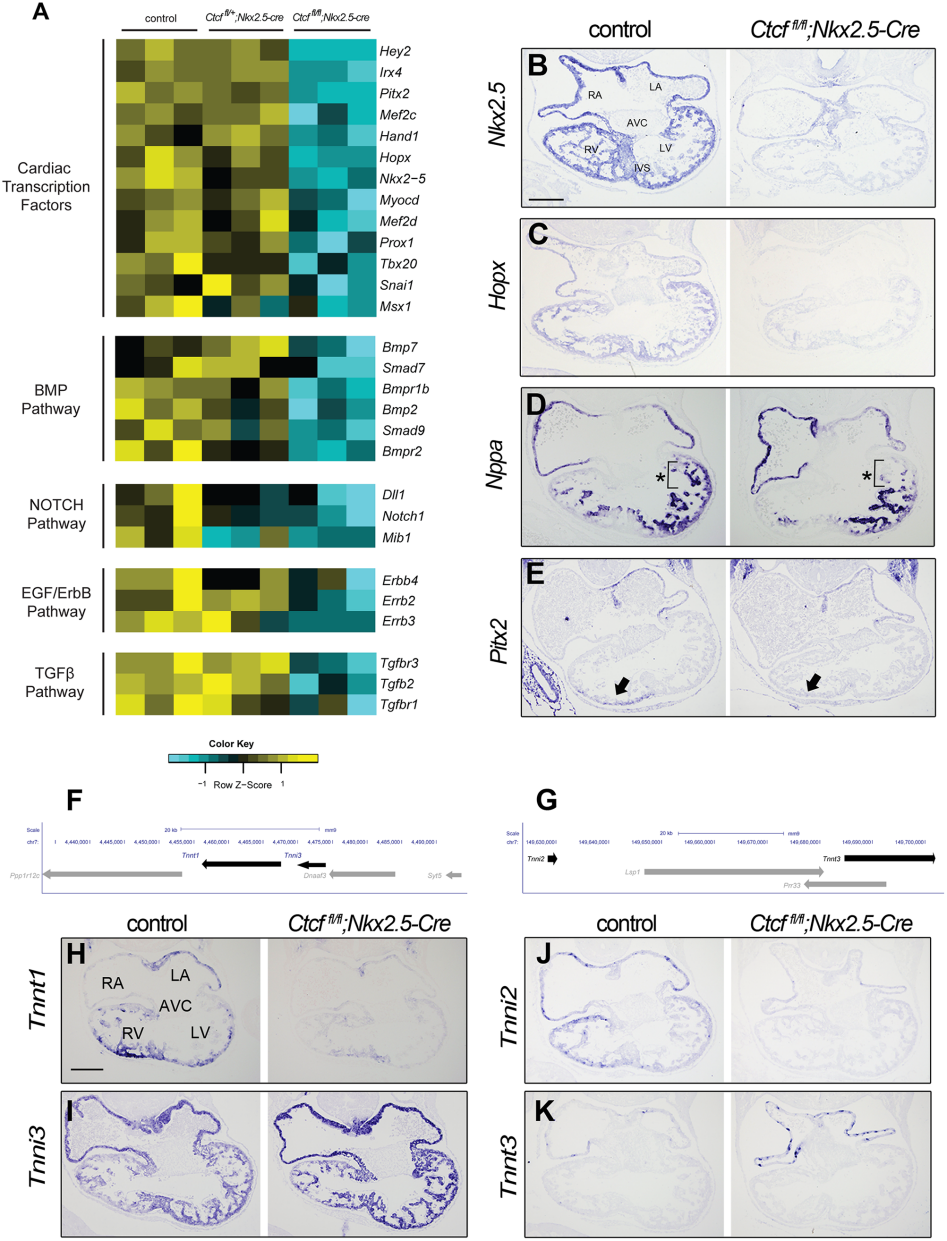
The cardiac developmental program is abrogated in *Ctcf* mutant hearts. (A) Deletion of *Ctcf* leads to downregulation of genes encoding key cardiac developmental transcription factors and signaling-pathway components. The heatmap shows normalized gene expression values from the RNA-seq data for controls (*Ctcf*^*fl/+*^), heterozygotes (*Ctcf*^*fl/+*^;*Nkx2*.*5-Cre*) and homozygotes (*Ctcf*^*fl/fl*^;*Nkx2*.*5-Cre*) across three biological replicates each. (B-E) *In situ* hybridization in sections of E10.5 control and mutant (*Ctcf*^*fl*/*fl*^;*Nkx2-5-Cre*) hearts for *Nkx2*.5 (B), *Hopx* (C), which are strongly downregulated; and *Nppa* (D) and *Pitx2* (E), which lose expression in the left ventricle (brackets and asterisks in D) or right ventricle (arrow in E), respectively. (F, G) Genomic region containing *Tnnt1* and *Tnni3* (F; mm9, chr7:4,433,080–4,493,651) or *Tnni2* and *Tnnt3* (G; mm9, chr7:149,623,608–149,703,181). Troponin genes are highlighted in black. (H-K) *In situ* hybridization in sections of E10.5 control and mutant hearts for *Tnnt1* (H), *Tnni3* (I), *Tnni2* (J) and *Tnnt3* (K). RA, right atria; LA, left atria; RV, right ventricle; LV, left ventricle; AVC, atrioventricular canal; IVS, interventricular septum. Scale bar, 200 μm.

The set of genes downregulated in *Ctcf* mutant hearts that are enriched in heart development functional categories include key transcription factors and members of several signaling pathways controlling cardiac development (Fig 4A). *In situ* hybridization confirmed downregulation of the transcription factors *Nkx2-5* and *Hopx* in the *Ctcf* mutant heart at E10.5. (Fig 4B and 4C). This analysis also showed reduced expression of *Nppa* to a more restricted domain in the left ventricle, and loss of *Pitx2* expression in the right ventricle (Fig 4D and 4E).

Our RNA-seq showed reciprocal up and downregulation of genes from the *Tnnt1*/*Tnni3* and *Tnni2*/*Tnnt3* troponin clusters (Fig 4F and 4G). Accordingly, *in situ* hybridization revealed strong downregulation of *Tnnt1* and *Tnni2*, and upregulation of *Tnni3* in atria and ventricles and of *Tnnt3* in atria in the *Ctcf* mutant heart (Fig 4H–4K). These changes in the expression pattern of troponin genes, which are arranged in clusters in the genome, suggest that CTCF coordinates their expression during development.

**Fig 5:**
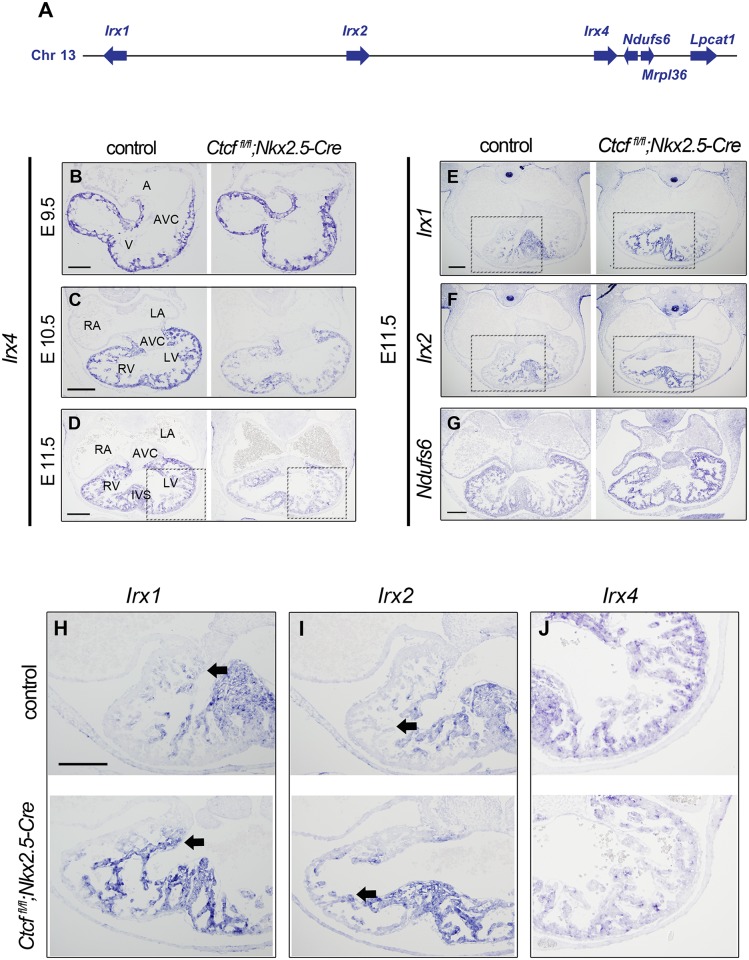
*Ctcf* deletion causes global changes in the expression of the *IrxA* cluster in the developing heart. (A) Diagram of the extended *IrxA* cluster showing the location of *Irx1*, *Irx2*, *Irx4* and the neighboring genes *Ndufs6*, *Mrpl36* and *Lpcat1* (not to scale). (B-J) *In situ* hybridization in sections of control and mutant hearts for *Irx4* at E9.5 (B), E10.5 (C) and E11.5 (D); and *Irx1* (E), *Irx2* (F) and *Ndufs6* (G) at E11.5. (H-I) Higher magnifications (black dashed boxes) of *Irx1* (H), *Irx2* (I) and *Irx4* (J) expression in control and mutant hearts at E11.5. Arrows indicate extension in the expression territories of *Irx1* (H) and *Irx2* (I). A,atria; V, ventricle, RA; right atria; LA, left atria; RV, right ventricle; LV, left ventricle; AVC, atrioventricular canal; IVS, interventricular septum. Scale bars, 100 μm (B), 200 μm (C-J).

*Irx4*, which encodes a transcription factor critical for heart development [34, 35] was downregulated in *Ctcf* mutants. We analyzed this gene and its genomic context in more detail as a means to understand the mechanisms through which CTCF regulates transcription *in viv*o. *Irx4* forms part of the 1.5 Mb *IrxA* cluster, which also contains the related *Irx1* and *Irx2* genes [36]. All three *IrxA* genes are expressed in the developing heart, in distinct but partially overlapping patterns. Whereas *Irx1* and *Irx2* express at low levels in the interventricular septum, *Irx4* is strongly expressed throughout the ventricles [37]. Previous studies have shown that the *IrxA* cluster is regulated through long-distance gene-specific interactions [38]. Furthermore, our RNA-seq analysis revealed significant upregulation of the three genes closest to *Irx4* outside the *IrxA* cluster: *Ndufs6*, encoding a subunit of mitochondrial Complex I; *Mrpl36*, encoding a mitochondrial ribosomal protein; and *Lpcat1*, involved in phospholipid metabolism (Fig 5A; S4 Table).

To confirm and extend these observations, we examined the expression of the three genes in the *IrxA* cluster, and of their immediate neighbor *Ndufs6*, in control and mutant hearts by *in situ* hybridization. Expression of *Irx4* in mutant hearts was indistinguishable from controls at E9.5 (Fig 5B), but expression levels were strongly reduced in mutants at E10.5 (Fig 5C) and E11.5 (Fig 5D and 5J). The RNA-seq analysis showed that *Irx1* and *Irx2* expression was not significantly different in mutants. However, *in situ* hybridization showed that their expression domains expanded from the interventricular septum to the adjacent trabecular myocardium in the right and left ventricle of *Ctcf* mutants (Fig 5E, 5F, 5H and 5I). In control hearts, *Ndufs6* was expressed ubiquitously but with higher intensity in the ventricles, similar to *Irx4*, but its expression was subtly increased in mutant hearts (Fig 5G). Together, these observations suggest that loss of CTCF leads to overall dysregulation of the *IrxA* cluster and its neighboring genes, perhaps through modification of its 3D structure.

### CTCF regulates local chromatin structure of the *IrxA* cluster

**Fig 6:**
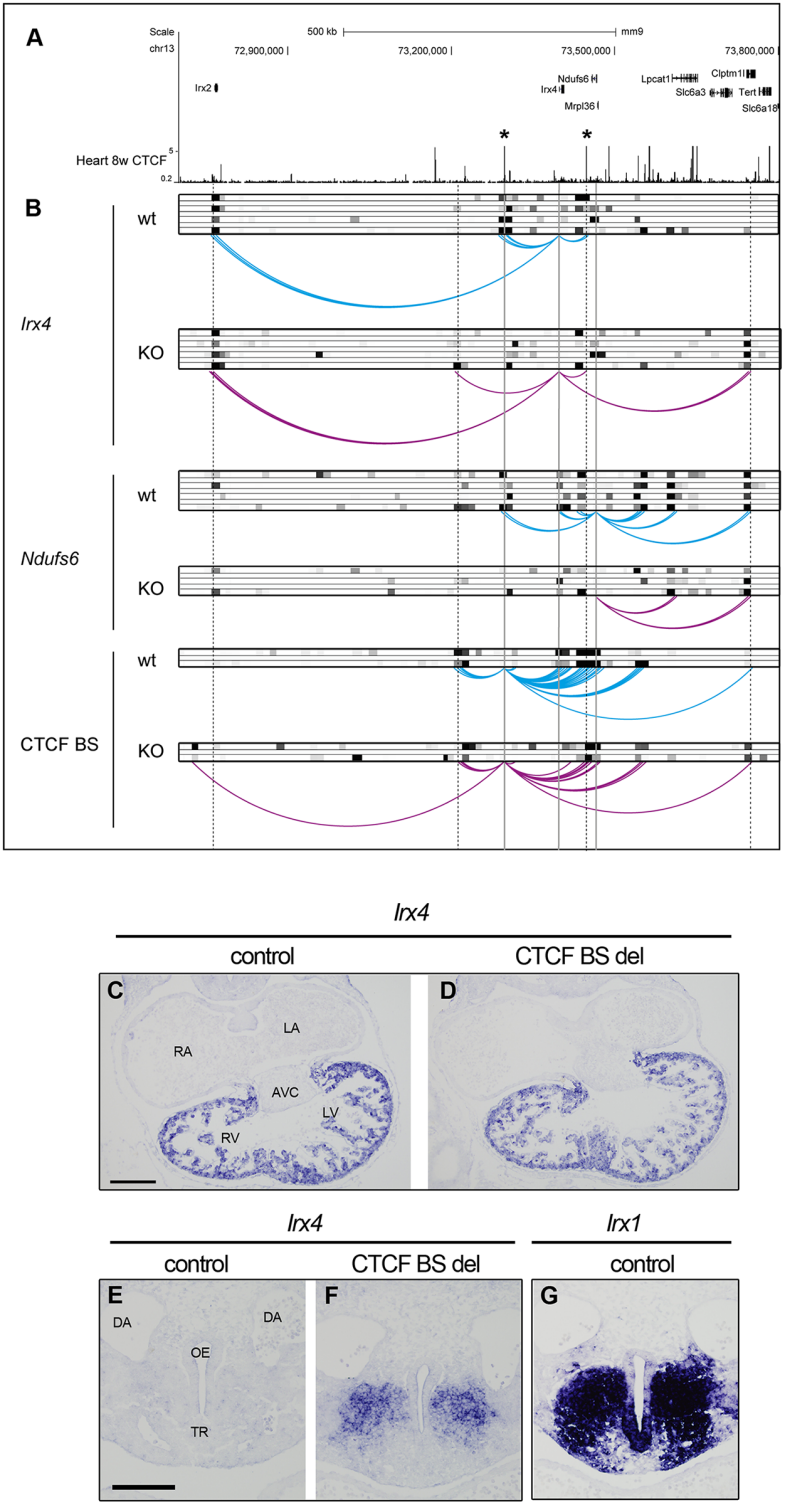
CTCF is necessary for the correct chromatin organization of the *Irx4*-*Ndufs6* locus. (A) ENCODE data for CTCF binding in 8 weeks mouse heart (mm9 chr13:72700492–73802866). Asterisks indicate specific intergenic CTCF binding sites. (B) Chromatin interaction profiles as determined by 4C-seq from the *Irx4* (top) and *Ndufs6* (middle) promoters and the *Irx2*/*Irx4* intergenic CTCF binding site (BS, bottom) in control (blue) and mutant (KO, purple) E11.5 hearts. Heat map of z-scores > 2 of replicates (grayscale) are shown on top of the interaction plots. (C-F) *Irx4 in situ* hybridization in E10.5 embryos from a transgenic mouse line where the *Irx2*/*Irx4* intergenic CTCF BS (left asterisk in A) has been deleted. (C, D) *Irx4* expression in the heart; (E, F) ectopic *Irx4* expression in the oral-esophageal region of foregut. (G) *Irx1* expression in the oral-esophageal region of foregut in a wild-type E10.5 embryo. RA, right atria; LA, left atria; RV, right ventricle; LV, left ventricle; AVC, atrioventricular canal; DA, dorsal aorta; TR, trachea; OE, oral-esophageal region. Scale bar, 200 μm (C, D), and 100 μm (E-G).

To uncover the function of CTCF in regulating the *IrxA* chromatin structure we performed chromosome conformation capture followed by deep sequencing (4C-seq) [39], using as viewpoints the promoters of *Irx4* and *Ndufs6* in E11.5 control and homozygous mutant (*Ctcf*^*fl/fl*^;*Nkx2*.*5-Cre*) hearts (Fig 6A and 6B; S7 Fig). In controls, the promoter of *Irx4* interacted strongly with the *Irx2* promoter and with specific CTCF binding sites located upstream and downstream in the *Irx2*/*Irx4* and *Irx4*/*Ndufs6* intergenic regions, respectively (asterisks in Fig 6A). The CTCF binding sites were previously identified by ChIP-seq in E14.5 and 8-week hearts [10]). In mutants, the interaction of the *Irx4* promoter with the *Irx2*/*Irx4* intergenic CTCF site was lost, and new interactions appeared upstream and 350 kb downstream in the *Clptm1l* locus (Fig 6B). The *Ndufs6* promoter established interactions with the promoters of *Irx4*, *Lpcat1* and *Clptm1l* as well as with the two intergenic CTCF sites in control hearts. In CTCF mutants, interactions of the *Ndufs6* promoter with both CTCF binding sites and the *Irx4* promoter were lost, and only contacts with the promoters of the downstream genes *Lpcat1* and *Clptm1l* were maintained (Fig 6B).

Finally, we used a viewpoint for 4C-seq the *Irx2*/*Irx4* intergenic CTCF site, which interacts with the promoters of *Irx4* and *Ndufs6* (Fig 6B, S7 Fig). In controls, the *Irx2*/*Irx4* intergenic CTCF site interacts upstream and downstream of and extended region spanning *Irx4*, *Ndufs6* and *Mrpl36*, and the downstream *Clptm1l* locus (Fig 6B). Interaction between the CTCF site and the *Irx4* promoter was greatly reduced in *Ctcf* mutants, reciprocating the pattern observed when using *Irx4* as viewpoint. Furthermore, novel interactions appeared that extend to *Irx2* (Fig 6B). Overall, these results suggest that CTCF plays a central role in organizing chromatin in the *Irx4* regulatory domain by mediating the interaction of several CTCF-bound sites with various promoters in the region. Removal of CTCF from the developing heart re-structures the local 3D organization of the extended *IrxA* cluster; this results in loss of the interaction between *Irx4* and *Ndufs6* with flanking CTCF sites. These results also suggest that CTCF limits chromatin interactions domains, as its loss causes an expansion of local contacts.

To unambiguously demonstrate that the CTCF binding site located in the *Irx2*/*Irx4* intergenic region is necessary for proper expression of *Irx4*, we generated a mouse line in which we deleted such CTCF binding site using the CRISPR/Cas9 system [40] (S8 Fig). Homozygote mice for the deletion are viable and fertile, as expected since *Irx4* mutant mice are also viable in homozygosity and only show mild hypertrophy and compromised contractility as adults [34]. We analyzed the expression of *Irx4* by *in situ* hybridization in E10.5 embryos from this line. *Irx4* was slightly reduced in the ventricles of the mutant line as compared with controls (Fig 6C and 6D). *Irx4* was also ectopically expressed in the oral-esophageal region (Fig 6E and 6F), in which *Irx1* is normally expressed (Fig 6G). Therefore, deletion of the *Irx2*/*Irx4* intergenic CTCF site leads to expansion of the expression domain of *Irx4*, perhaps by allowing *Irx1* regulatory elements to contact and activate *Irx4*. We have thus shown how CTCF is critical to maintain the correct chromatin structure across the *IrxA* cluster and neighboring genes, and that specific CTCF binding sites are essential for the proper regulation of gene expression.

## Discussion

Recent years have seen substantial advances in our understanding of the relationship between chromatin structure and gene expression. It is now clear that the spatial organization of the genome sets constraints that determine how different functional elements (promoters, enhancers, and boundaries) interact with one another [14]. Nevertheless, we still do not fully understand how this 3D structure is maintained or the role played by chromatin-bound factors in this process. In this study we have explored how one of these factors, CTCF, regulates gene expression and genome structure, by analyzing the effects of its loss during the development of the mammalian heart.

Deletion of *Ctcf* in the developing heart rapidly leads to cardiac defects and embryonic death. However, transcriptomic analysis shows that loss of CTCF does not lead to dysregulation of gene expression across large chromosomal domains; rather, changes appear to be local, as described in other developmental settings [20]. Upregulated and downregulated genes are both more likely to have CTCF binding sites in their vicinity, but only downregulated genes are closer to heart enhancers [10]. Downregulated genes include major regulators of the cardiac developmental program, strongly suggesting that CTCF facilitates enhancer-promoter interactions for these genes in a tissue-specific fashion. In contrast, upregulated genes are highly enriched for genes involved in mitochondrial function and protein translation, which interestingly show higher levels of CTCF binding close to their promoters. Cardiomyocyte maturation involves an increase in the demand for energy and protein production, which is accompanied by increased transcription of mitochondrial and ribosomal genes [8]. Cardiomyocytes lacking CTCF prematurely activate these programs, but fail to maintain functional mitochondria despite the increase in transcription. Therefore, we observe a premature maturation of cardiomyocytes lacking CTCF, accompanied by the shutting down of developmental and patterning processes. Nevertheless, there is a lack of coordination of this precocious differentiation, leading to embryonic lethality at E12.5.

To gain insight into the relationship between CTCF, genome structure and the regulation of gene expression, we analyzed the *IrxA* gene complex. This complex is a paradigm of gene clustering [36], with genes expressed in overlapping but distinct domains in the developing heart [37] and separated into distinct structural and regulatory domains [38]. We observed changes in the levels and pattern of *IrxA* gene expression in *Ctcf* mutants, and interestingly detected similar changes in unrelated genes neighboring *Irx4*. This strongly suggests that the changes in the extended *IrxA* cluster are caused by the loss of CTCF binding in the region, and are not a secondary effect of changes in other genes.

Analysis by 4C-seq in wild type and mutant hearts showed that when CTCF is lost, the *Irx4* promoter forms fewer contacts with CTCF-flanking sites and gains interactions with regions situated outside of its regulatory domain. Furthermore, the promoter of the mitochondrial *Ndufs6* gene also losses interactions with these CTCF sites. These changes in promoter interactions in *Ctcf* mutants were mirrored by 4C analysis of the *Irx2*/*Irx4* intergenic CTCF site. Consequently, deletion of this CTCF site leads to reduced *Irx4* cardiac expression accompanied by gain of expression in novel territories. Therefore, in this context CTCF is acting both as an insulator to separate adjacent regulatory domains, and as a facilitator of promoter-enhancer interactions to ensure proper gene expression within these domains.

The structure we have defined for *Irx4* is reminiscent of the recently described super-enhancer domains (SD), where regions of a few hundred kilobases located between CTCF sites are organized as insulated neighborhoods within large-scale topological domains [41]. The domain we identify would contain tissue specific enhancers together with their target genes. As is the case with SDs, loss of CTCF leads to downregulation of the gene central to this domain (*Irx4*) and upregulation of flanking genes (*Irx1* and *Irx2* on one side; *Ndufs6*, *Mrpl36*, and *Lpcat1* on the other).

Overall, our analysis suggests that chromatin structure is relatively stable during development and that the deletion of CTCF does not cause a marked disassembly of this organization, in line with recent reports [42]. However, local intra-TAD domains loops [16, 43] are affected, leading to dysregulation of genes important in the cardiac developmental program as we observe for the *IrxA* genes.

More puzzling is the observation that only a limited set of genes is altered by *Ctcf* deletion in the heart. CTCF binds to thousands of sites throughout the genome, most of which are shared between different tissues and cell lines [10, 12, 44]. An essential role for CTCF in determining and maintaining chromatin structure and organization was therefore anticipated [30]. Accordingly, constitutive loss of CTCF results in very early embryonic death [17–19] and its selective deletion in different developmental contexts leads to profound defects in the targeted organ or tissue, usually through increased apoptosis [20–22]. However, in all of these cases the same pattern is observed: despite wide distribution of CTCF binding, only a fraction of expressed genes is dysregulated. Furthermore, there is little overlap among genes regulated by CTCF in different systems, indicating a context-specific role of this factor. In the developing heart, we observe the concomitant upregulation and downregulation of maturation and developmental programs, suggesting that the role of CTCF here is to maintain the coordination of expression transitions.

In this scenario, only genes subject to dynamic regulation at the time of *Ctcf* deletion would show changes in expression. The description of different models of neural-specific deletion of *Ctcf* is compatible with this interpretation. When deleted in precursors during neural development, CTCF loss leads to apoptosis and subsequent death [21, 22]. However, deletion in postnatal neurons results in long-term survival of *Ctcf* mutant neurons, but activity-induced changes of gene expression are altered [45]. Together, these observations suggest that CTCF, and possibly local chromatin structure, are not necessary for basal gene activity but essential for dynamic transitions in expression.

## Materials and methods

### Ethics statement

Mice were bred in the core animal facility in the Centro Nacional de Investigaciones Cardiovasculares in accordance with national and European legislation. All procedures were approved by the CNIC Committee of Animal Welfare and by the Madrid Autonomous Government Regional Ministry of the Environment and Territorial Organisation (reference number PROEX 196/14).

### Mouse strains

The *Ctcf* floxed allele and *Nkx2*.*5*-*Cre* line have been previously described [17, 23]. Primers used for genotyping are detailed in S8 Table. *Ctcf*^*fl/+*^ or *Ctcf*^*fl/fl*^ embryos were used as controls. Mice were bred in the core animal facility in the Centro Nacional de Investigaciones Cardiovasculares in accordance with national and European legislation. Some of the experiments were performed in Toronto, and they were approved by the Toronto Centre for Phenogenomics Animal Care Committee.

### Histology and immunohistochemistry

Whole mount embryos were dissected in cold PBS and imaged using a Nikon SMZ1500 microscope with NIS-Elements BR 4.12.01 imaging software. For sections, embryos were collected in cold PBS and fixed in 4% PFA overnight at 4°C, dehydrated in an ethanol series, embedded in paraffin, and sectioned at 5 μm for immunostaining amd hematoxylin and eosin, and at 7 μm for *in situ* hybridization. Sections were observed under an Olympus BX51 microscope and photographed with an Olympus DP71 digital camera. 5 μm paraffin sections were incubated with CTCF 1:1500 (Bethyl labs A300-543A) and CT3 1:10 (Hybridoma Bank) or processed for histological analysis by hematoxylin and eosin staining. For TUNEL the Terminal Transferase recombinant kit (Roche 03 333 574 001) and biotin-16-dUTP (Roche 11 093 070 910) were used, together with anti-PH3 antibody 1:200 (Millipore 06–570) and CT3 1:10 (Hybridoma Bank); images were acquired with a Nikon A1R Confocal microscope.

Quantification of TUNEL and PH3 staining were performed with ImageJ software. Positive cells for each antibody were counted in 3–5 sections per heart. Three control and three KO hearts were used. Statistical significance was determined using one-tailed Student´s test. α-actinin bands quantification was performed as previously described [3]. Quantification of CTCF staining at E9.5 was performed with ImageJ software. Positive cells for CTCF were counted in 5 sections per heart.

For cardiomyocyte purification, individual E10.5 ventricles were dissected in cold PBS and incubated 5 min at 37°C with 100 μL of 48 mg/mL Collagenase type II and pancreatin 0.025gr/mL (plus NaCl 0.0085gr/mL) diluted in ADS buffer (5x: 0.034 g/mL NaCl, 0.238 g/mL Hepes, 0.0006 g/mL NH_2_PO_4_, 0.005 g/mL glucose, 0.002 KCl, 0.001 g/mL MgSO_4_). After mechanical disruption by pipetting, tubes were left 2 min in the hood, liquid phase was separated, mixed with 100microL of cardiomyocyte media (4:1 mixture of of Dulbecco’s Modified Eagle Medium (DMEM, 1x Gibco, 41965–039) and medium M199 (Sigma, M2154). This is supplemented with 15% inactivated bovine serum; 5mM HEPES, pH7.4; 2mM L-glutamine; and 1x penicillin-streptomycin [46] and incubated at 37°C while enzymatic and mechanical disruption was repeated. Both fractions were pooled and centrifuged at 1000 rpm at room temperature. Pellets were resuspended in 300 μL of cardiomyocyte culture media and incubated 45 min in a 24 well plate previously coated with gelatin 1% at 37°C. Cardiomyocytes were counted and incubated in a new 24 well plate without gelatin for 24 hrs. Cardiomyocytes were fixed with PFA diluted in cardiomyocyte media (4% for α-actinin sarcomeric staining, 3.6% for Tom20 staining) for 10 min at 4°C, washed 2x with PBS and stored at 4°C until used. Cardiomyocytes were blocked with BSA, and incubated ON 4°C with 1:100 Tom20 or α-actinin.

### *In situ* hybridization

*In situ* hybridization using digoxigenin-labeled probes (Roche 11277073910) was performed as described [47] and developed with anti-dioxigenin-AP (Roche 11093274910) and BM-Purple (Roche 11442 074 001). Probes for *Irx1* and *Irx2* were previously described [48]. Probes for *Nkx2*.5, *Nppa* and *Pitx2* were kindly provided by Jose Luis de la Pompa (CNIC). All other probes were generated by PCR (primers used are detailed in S8 Table), adding the sequence of the SP6 promoter at the 5’ of forward primers and of the T7 promoter at the 5’ of reverse primers. Sections were observed under an Olympus BX51 microscope and photographed with an Olympus DP71 digital camera.

### RNA-seq analysis

RNA-seq was conducted on three pools of six E10.5 hearts each from control (*Ctcf*^*fl/+*^), heterozygotes (*Ctcf*^*fl/+*^;*Nkx2*.*5-Cre*) and homozygous mutants (*Ctcf*^*fl/fl*^;*Nkx2*.*5-Cre*). Sequencing was performed by the CNIC Genomics Unit using the GAIIx sequencer, and differential gene expression analysis was performed by the CNIC Bioinformatics Unit using the transcriptome set from Mouse Genome Reference NCBIM37 and Ensembl Gene Build version 65. GO term enrichment was done using DAVID (http://david.abcc.ncifcrf.gov) with cut-offs of minimum 10 genes and an EASE value of 0.0001 [28, 49]. Distances between transcriptional start sites (TSS) and between these and CTCF binding sites or predicted heart enhancers were analyzed using R. We used ChIP-seq data for CTCF in adult 8-week hearts. Although data is also available for E14.5 embryonic hearts, its quality is much lower (one replicate with 2.8M reads for E14.5; two replicates with over 8M reads each for 8w). Coordinates for CTCF binding sites, and predicted heart enhancers were obtained from the Ren Lab (http://chromosome.sdsc.edu/mouse/download.html) [10]). Heat maps were generated using R and the heatmap.2 package. For density plots of CTCF ChIP signal, values +/- 2kb relative to the TSS were extracted with bwtools [50] from bigWig files available at ENCODE (ENCSR000CBI), and z-scores were calculated transforming each value by substracting the mean over each chromosome and dividing by the standard deviation. After a smoothing step results were plotted using in-house scripts in R. The observed distances between TSS of gene-sets were compared with the expected proportion calculated from 5,000 groups randomly sampled without replacement taken from all expressed genes. To identify heart-specific CTCF sites, we first merged all sites identified across multiple tissues from the ENCODE data (121,147 sites) and subtracted these from the set of sites bound in heart. This analysis rendered only 1,432 heart-specific peaks that were not enriched near different sets of deregulated genes in the *Ctcf* mutant.

For known motif enrichment and *de novo* motif search using Homer [51], we defined the promoter-proximal region as the 200 bp immediately upstream from the TSS of differential-expressed genes belonging to each of the “development”, “translation” and “mitochondria” categories, and the distal region as the next 1.8 kb upstream and the 2 kb downstream of the TSSs. We then identified all CTCF peaks present in these regions for each category, and used the 200 bp sequences surrounding the summit of CTCF peaks as input. As a background, we used 2 kb upstream of the TSSs of genes expressed but unchanged in our RNAseq data that had no CTCF peaks in that region (8,445 genes). As cut-offs we used p-value < 1e-10 for de novo motif search, and a Benjamini adjusted p-value < 0,001 for known motif enrichment.

All sequencing data has been deposited at GEO under accession number GSE77644.

### Western blot analysis

Hearts lysates of E10.5 and E11.5 prepared in RIPA buffer were separated by 12.5% SDS polyacrylaimde gel electrophoresis (SDS PAGE) and electroblotted onto PVDF transfer membrane (BioRad). For protein detection, the following antibodies were used: CoI (Complex IV subunit I; Invitrogen), Tom20 (Santa Cruz Biotech), αActin (Sigma Aldrich), CoxIV (Complex IV subunit IV), Grp75 and mtFA (Abcam). Quantification of signals arising from near-infrared (NIR) fluoropnores conjugated to secondary antibodies was performed by two-channel infrared (IR) scanner (Odyssey v.3.0). Representative gels are shown in figures (n = 3–4).

### Blue native gel electrophoresis

Mitochondrial isolation from E10.5 and E11.5 hearts were carried out in duplicate, as described [52] with some modifications. Pools of 4 embryonic hearts were homogenized in a glass homogenizer in buffer A (Sucrose 0,32M; Tris 10mM, EDTA 1mM; pH7,4). After centrifugation at 1000g, the nuclei were discarded and mitochondria were collected from the supernatant by a new centrifugation at 12000g. Mitochondrial proteins were solubilized with digitonin (4g/g) (Sigma D5628) and run on a 3%–13% gradient Blue Native gel as described [53]. After electrophoresis, the complexes were electroblotted onto PVDF membrane (Merckmillipore, IPFL00010) and sequentially probed with specific antibodies against Complex I (anti-Ndufa9, ab14713), Complex III (anti-core1, ab110252), Complex IV (anti-CoI, Invitrogen ref.459600), ATPsyntase (anti-ATPB, ab14730) and VDAC1 (anti-VDA1C, ab14734).

### Transmission electron microscopy (TEM)

Embryonic hearts from E10.5 and E11.5 were isolated from the specified genotype (n = 3). Samples were fixed in 2.5% glutaraldehyde (Sigma-Aldrich), 4% formaldehyde (Electron Microscopy Sciences) in 0.1M HEPES buffer for 3–4 h at 4°C, and processed as previously described [54]. Briefly, after buffer washes samples were post-fixed for 1h at room temperature in a 1:1 solution of 2% osmium tetroxide (Electron Microscopy Sciences) and 3% aqueous potassium ferrocyanide (Sigma-Aldrich). Samples were rinsed in distilled water. Tissues were dehydrated through a graded acetone series and embedded in Spurr's Low Viscosity embedding mixture (Electron Microscopy Sciences). Ultrathin sections (60 nm) were then mounted on copper grids and stained with lead citrate. Samples were examined on a JEOL 10–10 electron microscope and analyzed by ImageJ v.1.6.0 Software.

### 4C-sequencing

4C was performed as previously described [55, 56]. Briefly, hearts were dissected from control and mutant (*Ctcf*^*fl/fl*^;*Nkx2*.*5-Cre*) E11.5 embryos (in order to be able to obtain more starting material). Each heart was minced and passed throw a 70 micrometers cell strainer, crosslinked with 2% PFA, frozen with liquid nitrogen, and stored at -80° for later processing once genotypes were established. Pools of 45–65 hearts from controls or mutants were lysed and digested first with *Dpn*II (New England Biolabs, cat.no. R0543M) followed by *Csp*6I (Fermentas, cat.no. ER0211). For all experiments, 100 to 200 ng of the resulting 4C template was used for the subsequent PCR reaction (primers used are detailed in S8 Table). Sequencing was performed by the CNIC Genomics Unit. All sequencing data has been deposited at GEO under accession number GSE77644.

### 4C-seq data analysis

For sample comparison between controls and mutants, 4C-seq data was normalized by total weight in a window of 15 Mb surrounding the *IrxA* cluster. The mean and standard deviation for each fragment were calculated for each group of replicates, and the difference was determined between the means in control and mutants for each viewpoint. Data were compared using the R package edgeR [57], which applies statistical tests based on negative binomial distributions.

For contact estimation, aligned reads were assigned to their corresponding virtual first cutter digested genome fragment. Each fragment end was considered as a captured site (ligated to the viewpoint) if one or more sequences mapped to it starting at the end of the fragment and in the right orientation, facing the center of the fragment. The number of captured sites was summarized per 30 fragment window (max of 60 captured sites per window). The frequency of captured sites per window was used to fit a distance decreasing monotone function and z-scores were calculated from its residuals using a modified version of FourCSeq [58]. Significant contacts were considered in cases where the z-score was >2 in both replicates and deviated significantly (adjusted p value <0.05) from its normal cumulative distribution in at least one of the replicates.

### CRISPR/Cas9 genomic deletions

Two guide RNAs (g5.3-g3; S8 Table), together with the Cas9 protein with NLS (PNA Bio), were injected into the pronucleus of E0.5 B6/CBA F1 embryos, which were then transferred to CD1 pseudo-pregnant females. Guide RNAs were injected at 25ng-μL, and the Cas9 protein at 30 ng/μL. Embryos from the established line were collected at E10.5 and processed for *in situ* hybridization and genotyping (primers listed in S8 Table).

## Supporting information

S1 Fig *Nkx2*.*5-Cre*/+ and *Ctcf*^*fl/+*^;*Nkx2*.*5-Cre*/+ heterozygotes show no cardiac defects. Hematoxylin and eosin staining at E9.5 (A-F), E10.5 (G-L), and E11.5 (M-R) of control (A, B, G, H, M, N), *Ctcf*^*fl/+*^;*Nkx2*.*5-Cre* (C, D, I, J, O, P) and *Nkx2*.*5-Cre* heterozygous embryos (E, F, K, L, Q, R). Higher magnifications (black dashed boxes) for each section are shown below. A, atria; V, ventricle; AVC, atrioventricular canal; RA, right atria; RV, right ventricle; LA, left atria; RA, right ventricle; IVS, interventricular septum. Scale bars, 100 μm (A, G, M) and 200 μm (B, H, N).
(TIF)

S2 Fig CTCF is lost at E10.5 in *Ctcf* ^*fl/fl*^;*Nkx2*.*5-Cre* mutant hearts. Immunofluorescence for CTCF (red) and cardiac troponin T (CT3, green) in sections of control and mutant hearts (*Ctcf* ^*fl/fl*^;*Nkx2*.*5-Cre*) at E9.5 (A, B), E10.5 (C, D) and E11.5 (E, F). Higher magnifications (white dashed boxes) of each section are shown below. Arrowheads point to CT3 positive cardiomyocytes show that loose CTCF signal in the mutants in comparison with the control. Arrows point to endocardial cells that express CTCF at comparable levels in mutant and control hearts. A, atria; V, ventricle; RA, right atria; LA, left atria; RV, right ventricle; LV, left ventricle; AVC, atrioventricular canal; IVS, interventricular septum. Scale bars, 100 μm (E9.5, higher magnification of E10.5) or 200 μm (E10.5, E11.5).
(TIF)

S3 Fig CTCF loss does not affect apoptosis or proliferation in the developing heart. Cell death (A-F) and proliferation (G-L) in control and mutant (*Ctcf*^*fl*/fl^;*Nkx2-5-Cre*) hearts at E10.5. Cardiomyocytes are labelled with CT3 antibody (white; A, B, G, H), TUNEl (green (C, D) or phosphohistone 3 antibody (red, I, J). Arrowheads in merged images (E, F, K, L) point to positive nuclei. Quantifications of TUNEL and PH3 in cardiomyocytes at E10.5 (M, N) and E11.5 (O, P) showed no significant difference. RA, right atria; LA, left atria; RV, right ventricle; LV, left ventricle; AVC, atrioventricular canal. Scale bar, 200 μm.
(TIF)

S4 Fig Functional annotation of genes dysregulated by *Ctcf* deletion in the developing heart. Detailed GO term enrichment in gene dysregulated in cardiac *Ctcf* mutants related to mitochondria (A) and translation (B).
(TIF)

S5 Fig Chromosomal distribution and genomic features of genes dysregulated by *Ctcf* deletion in the developing heart. (A) Distances between differentially regulated (brown), upregulated (yellow) or downregulated (blue) genes in *Ctcf* mutants. Dashed lines show the distribution of random permutation tests. (B) Distribution of distances from the nearest CTCF binding site (left panel) or heart enhancer (right panel) to the transcriptional start sites (TSS) of downregulated, upregulated, and expressed but unchanged genes in *Ctcf* mutant hearts. ** p < 10e-10 versus expressed but unchanged genes; Mann-Withney test.
(TIF)

S6 Fig Cellular phenotypes of *Ctcf* mutant cardiomyocytes. (A) Transmission Electron Microscopy showing balloning of mitochondria from mutant E11.5 cardiomyocytes that also have poorly organized crests. Scale bar, 1 μm. (B, C) Immunofluorecsence for Tom20 (B) and for sarcomeric α-actinin (C) in E10.5 control and mutant cardiomyocytes. Scale bars 20 μm (B-C). (D) Quantification of α-actinin bands per cell at E10.5 in control and mutant cardiomyocytes ns, not significant.
(TIF)

S7 Fig 4C-seq analysis of the *Irx4*-*Ndufs6* locus in control and *Ctcf* ^*fl/fl*^;*Nkx2*.*5-Cre* mutant hearts. (A) ENCODE data for CTCF binding in 8 weeks mouse heart (mm9 chr13:72700492–73802866). (B) 4C-seq profiles using viewpoints from the *Irx4* (red) and *Ndufs6* (green) promoters, and the *Irx2*/*Irx4* intergenic CTCF BS (blue) in control and mutant (KO) E11.5 hearts. Dark shading depicts the mean interaction profile; lighter shading represents the standard deviation of replicates. Arrowheads indicate the location of the viewpoint.
(TIF)

S8 Fig CRISPR/Cas9 mediated deletion of the *Irx2-Irx4* intergenic CTCF bidning site. (A) Top, ENCODE data for CTCF binding in 8 weeks mouse heart (mm9 chr13:72700492–73802866). The *Irx2*/*Irx4* intergenic CTCF BS is highlighted in green. Bottom, schematic representation of the location of the guide-RNAs used for genome editing of the CTCF BS. (B) Sequence of the wild type (wt) and deleted (del) alleles of the CTCF BS deleted. In red are the gRNAs sequences are indicate in red, and PAM sequences in bold lettering. (C) PCR genotyping of the different genotypes from the CTCF BS deleted mouse line.
(TIF)

S1 Table Genotypes of live embryos obtained from ♂*Ctcf*^*fl/+*^;*Nkx2*.*5-Cre*^*tg/+*^ X ♀*Ctcf*^*fl/fl*^ crosses. (PDF)

S2 Table Quantification of double positive CT3-CTCF nuclei in E9.5 mutant hearts. (PDF)

S3 Table Quantification of proliferation and apoptosis in E10.5-E11.5 control and mutant hearts. (PDF)

S4 Table RNA-seq analysis of *Ctcf*^*fl/+*^, *Ctcf*^*fl/+*^;*Nkx2*.*5-Cre* and *Ctcf*^*fl/fl*^;*Nkx2*.*5-Cre* E10.5 hearts. (XLSX)

S5 Table Gene ontology analysis of differentially expressed genes between E10.5 control and mutant hearts. (XLSX)

S6 Table Genes and functional annotations used to build Fig 2A and S3A and S3B Fig. (XLSX)

S7 Table Motif discovery in proximal and distal CTCF peaks of dysregulated genes in *Ctcf*^*fl/fl*^;*Nkx2*.*5-Cre*^*tg/+*^ E10.5 hearts. (PDF)

S8 Table Primers and oligonucleotides used in this study. (PDF)
