# Supplementary figures and images for: CTCF counter-regulates cardiomyocyte development and maturation programs in the embryonic heart
Source: PLoS Genet. 2017 Aug 28;13(8):e1006985. doi: 10.1371/journal.pgen.1006985 (PMC5591014; doi:10.1371/journal.pgen.1006985)

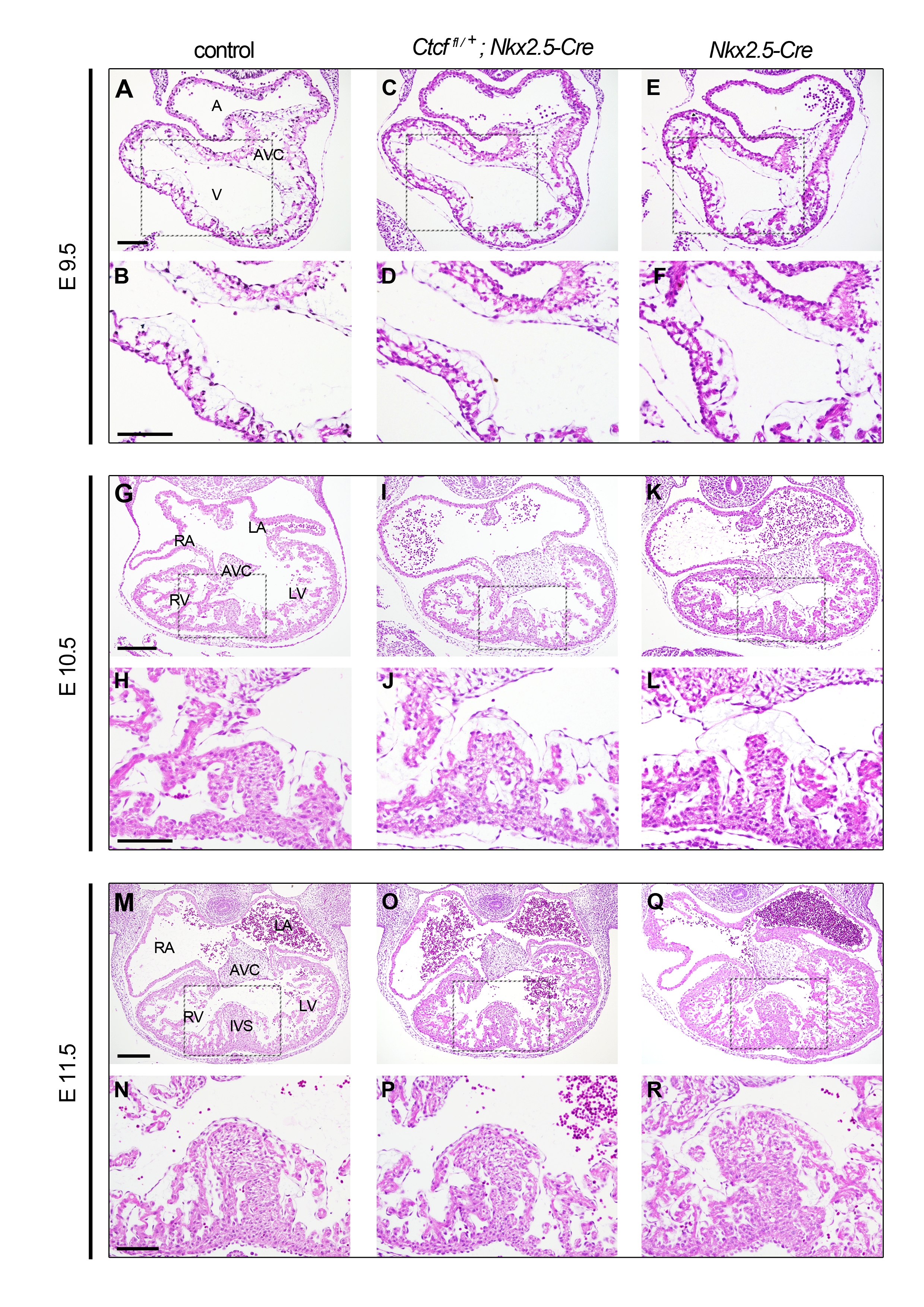

Supplement: S1 Fig — Hematoxylin and eosin staining at E9.5 (A-F), E10.5 (G-L), and E11.5 (M-R) of control (A, B, G, H, M, N), Ctcffl/+;Nkx2.5-Cre (C, D, I, J, O, P) and Nkx2.5-Cre heterozygous embryos (E, F, K, L, Q, R). Higher magnifications (black dashed boxes) for each section are shown below. A, atria; V, ventricle; AVC, atrioventricular canal; RA, right atria; RV, right ventricle; LA, left atria; RA, right ventricle; IVS, interventricular septum. Scale bars, 100 μm (A, G, M) and 200 μm (B, H, N). (TIF) [file pgen.1006985.s001.tif]

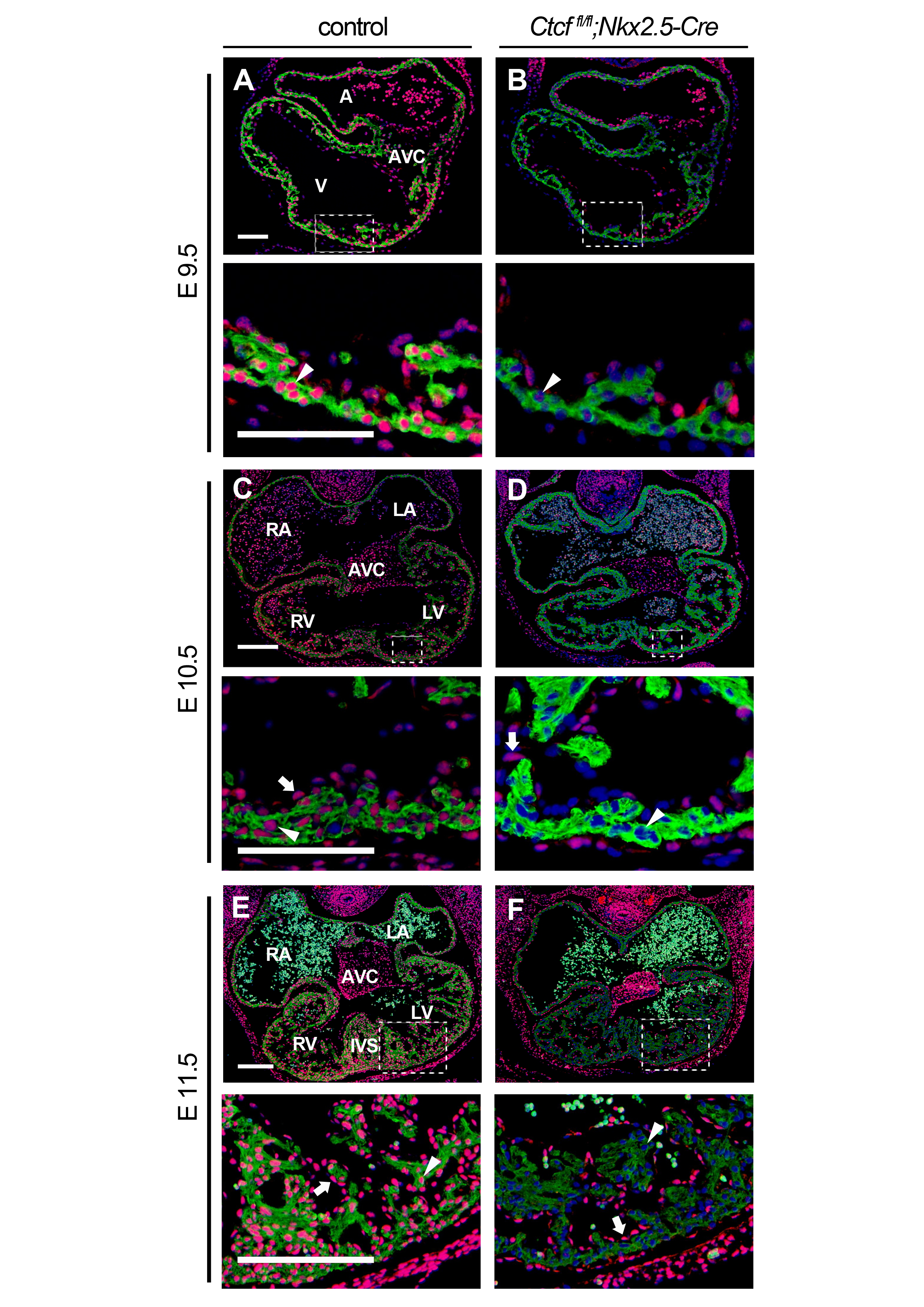

Supplement: S2 Fig — Immunofluorescence for CTCF (red) and cardiac troponin T (CT3, green) in sections of control and mutant hearts (Ctcf fl/fl;Nkx2.5-Cre) at E9.5 (A, B), E10.5 (C, D) and E11.5 (E, F). Higher magnifications (white dashed boxes) of each section are shown below. Arrowheads point to CT3 positive cardiomyocytes show that loose CTCF signal in the mutants in comparison with the control. Arrows point to endocardial cells that express CTCF at comparable levels in mutant and control hearts. A, atria; V, ventricle; RA, right atria; LA, left atria; RV, right ventricle; LV, left ventricle; AVC, atrioventricular canal; IVS, interventricular septum. Scale bars, 100 μm (E9.5, higher magnification of E10.5) or 200 μm (E10.5, E11.5). (TIF) [file pgen.1006985.s002.tif]

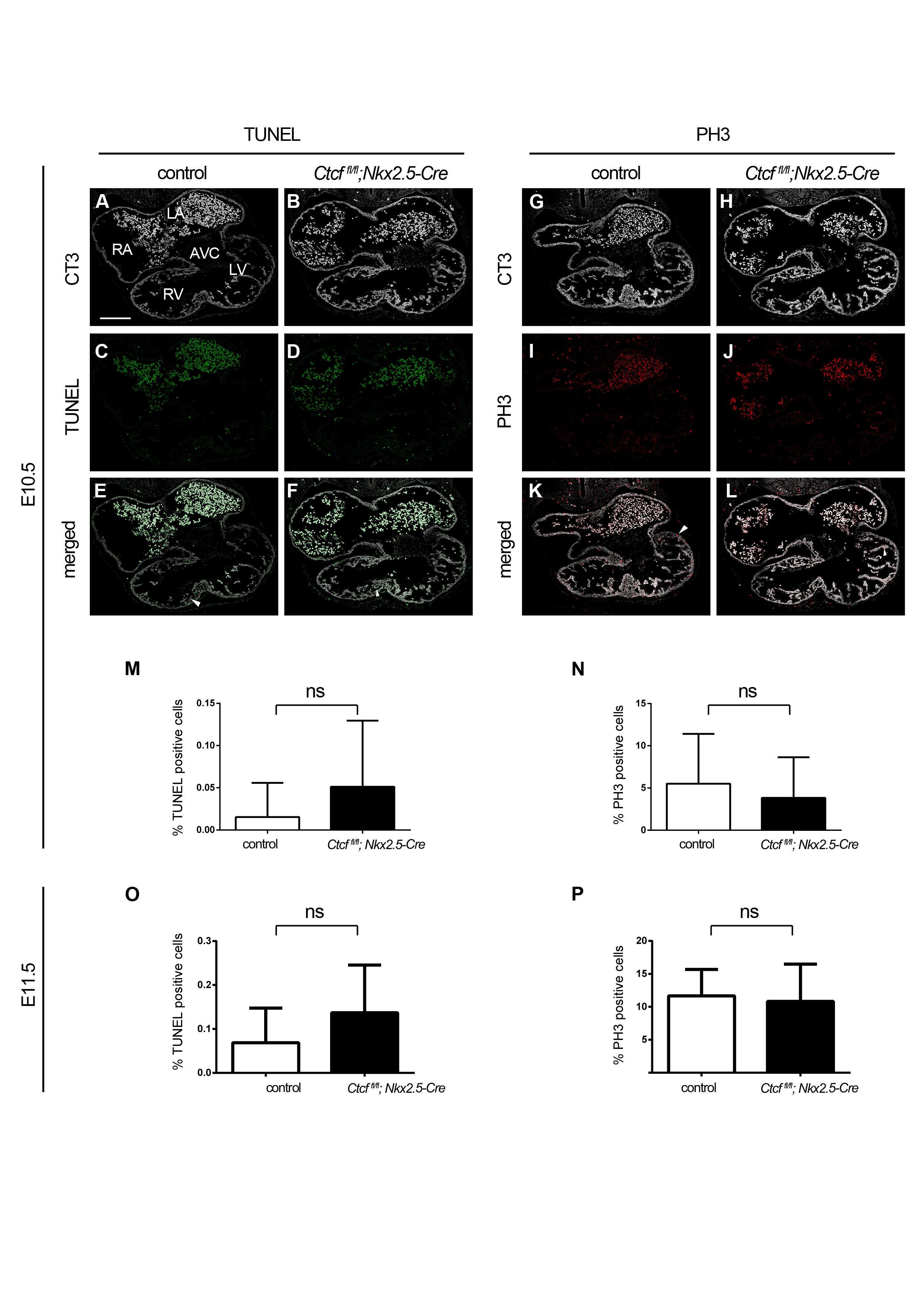

Supplement: S3 Fig — Cell death (A-F) and proliferation (G-L) in control and mutant (Ctcffl/fl;Nkx2-5-Cre) hearts at E10.5. Cardiomyocytes are labelled with CT3 antibody (white; A, B, G, H), TUNEl (green (C, D) or phosphohistone 3 antibody (red, I, J). Arrowheads in merged images (E, F, K, L) point to positive nuclei. Quantifications of TUNEL and PH3 in cardiomyocytes at E10.5 (M, N) and E11.5 (O, P) showed no significant difference. RA, right atria; LA, left atria; RV, right ventricle; LV, left ventricle; AVC, atrioventricular canal. Scale bar, 200 μm. (TIF) [file pgen.1006985.s003.tif]

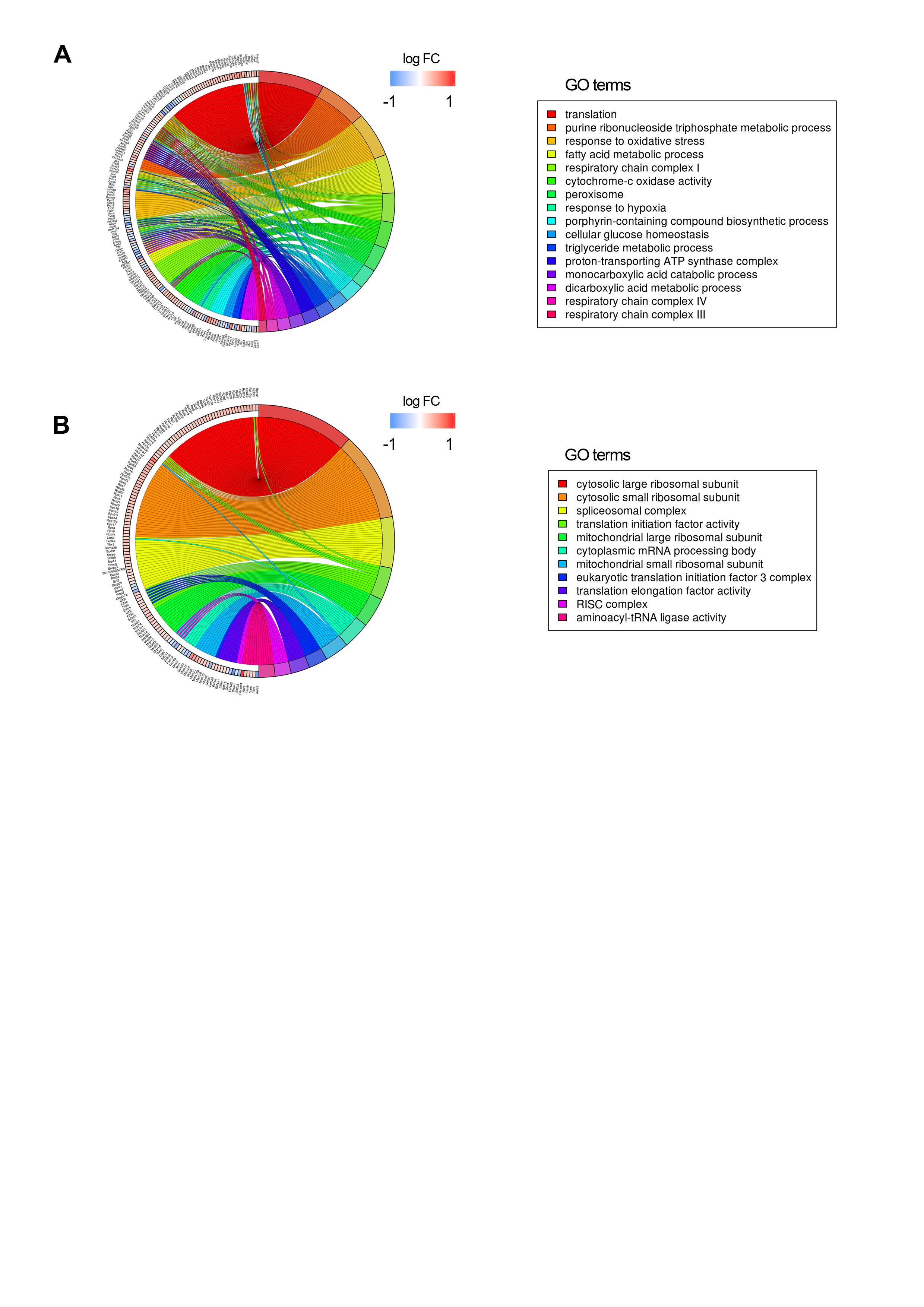

Supplement: S4 Fig — Detailed GO term enrichment in gene dysregulated in cardiac Ctcf mutants related to mitochondria (A) and translation (B). (TIF) [file pgen.1006985.s004.tif]

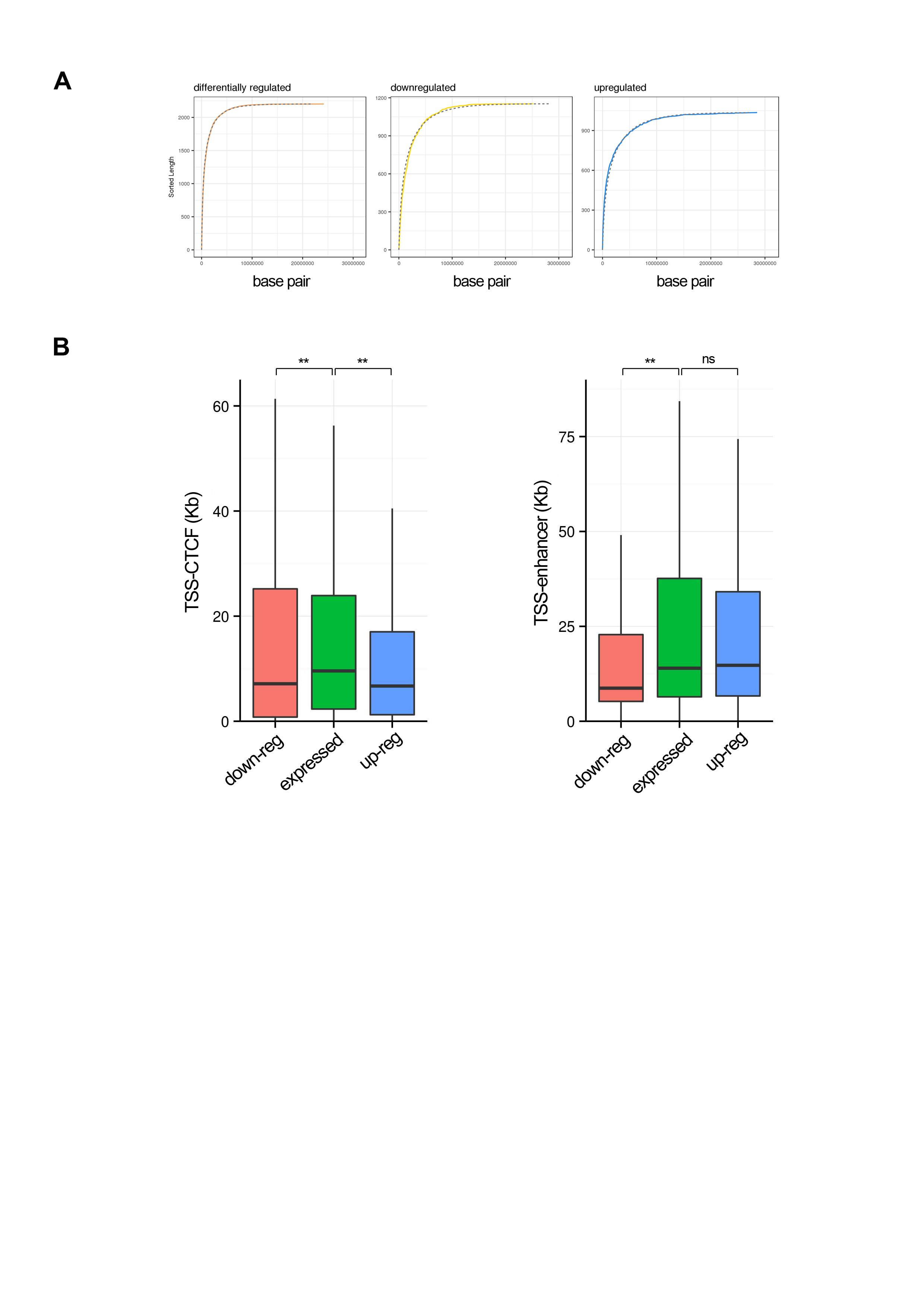

Supplement: S5 Fig — (A) Distances between differentially regulated (brown), upregulated (yellow) or downregulated (blue) genes in Ctcf mutants. Dashed lines show the distribution of random permutation tests. (B) Distribution of distances from the nearest CTCF binding site (left panel) or heart enhancer (right panel) to the transcriptional start sites (TSS) of downregulated, upregulated, and expressed but unchanged genes in Ctcf mutant hearts. ** p < 10e-10 versus expressed but unchanged genes; Mann-Withney test. (TIF) [file pgen.1006985.s005.tif]

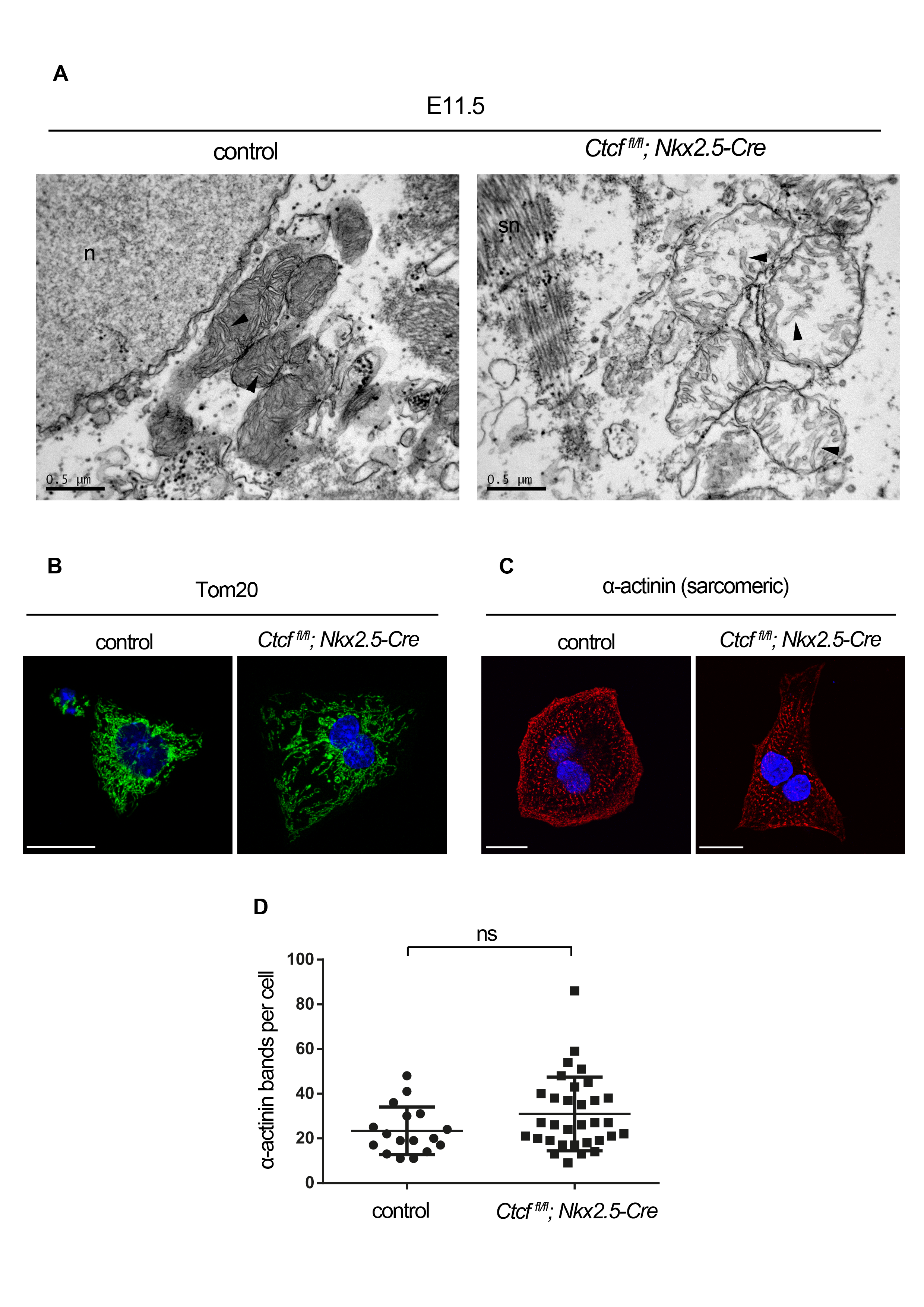

Supplement: S6 Fig — (A) Transmission Electron Microscopy showing balloning of mitochondria from mutant E11.5 cardiomyocytes that also have poorly organized crests. Scale bar, 1 μm. (B, C) Immunofluorecsence for Tom20 (B) and for sarcomeric α-actinin (C) in E10.5 control and mutant cardiomyocytes. Scale bars 20 μm (B-C). (D) Quantification of α-actinin bands per cell at E10.5 in control and mutant cardiomyocytes ns, not significant. (TIF) [file pgen.1006985.s006.tif]

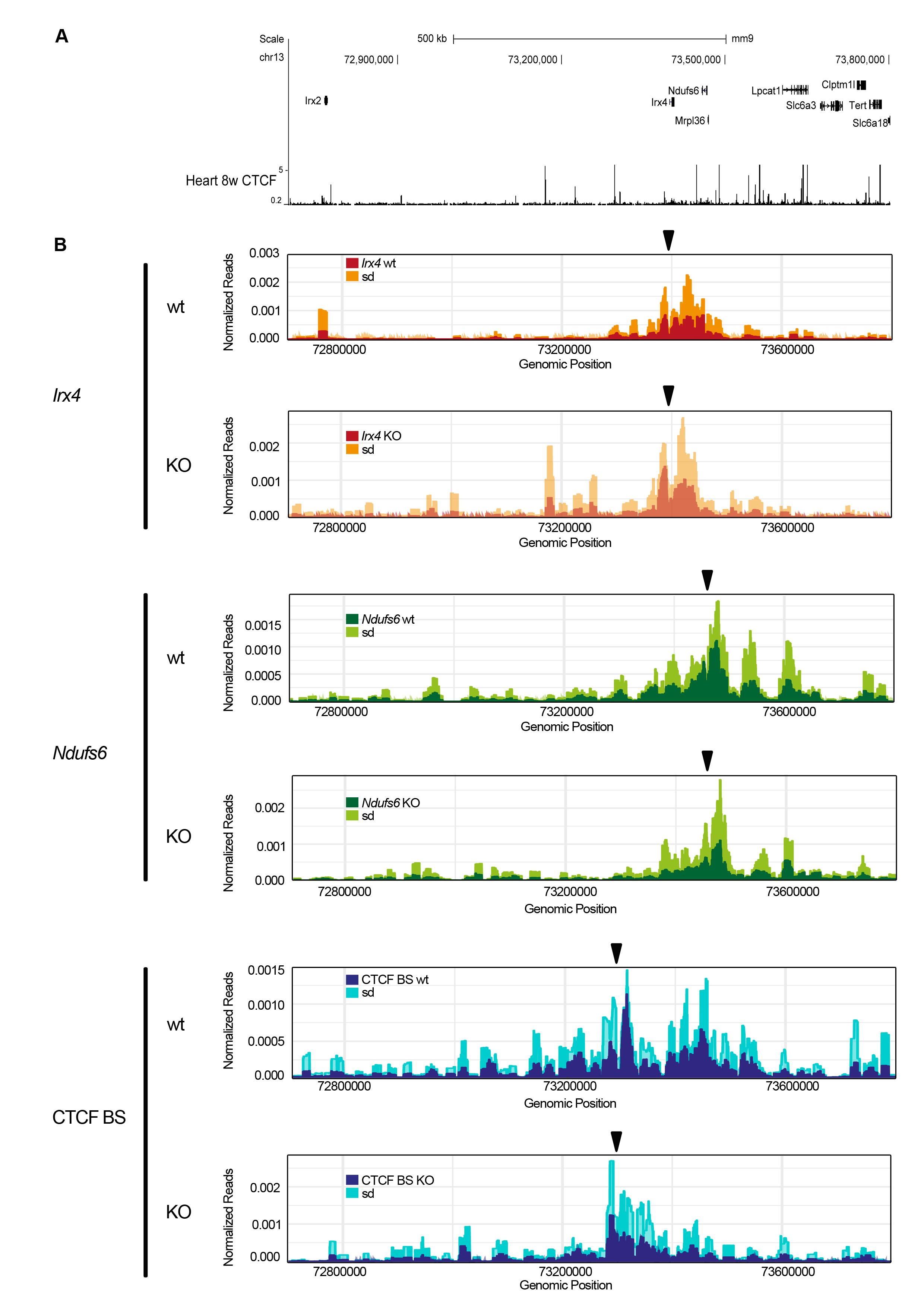

Supplement: S7 Fig — (A) ENCODE data for CTCF binding in 8 weeks mouse heart (mm9 chr13:72700492–73802866). (B) 4C-seq profiles using viewpoints from the Irx4 (red) and Ndufs6 (green) promoters, and the Irx2/Irx4 intergenic CTCF BS (blue) in control and mutant (KO) E11.5 hearts. Dark shading depicts the mean interaction profile; lighter shading represents the standard deviation of replicates. Arrowheads indicate the location of the viewpoint. (TIF) [file pgen.1006985.s007.tif]

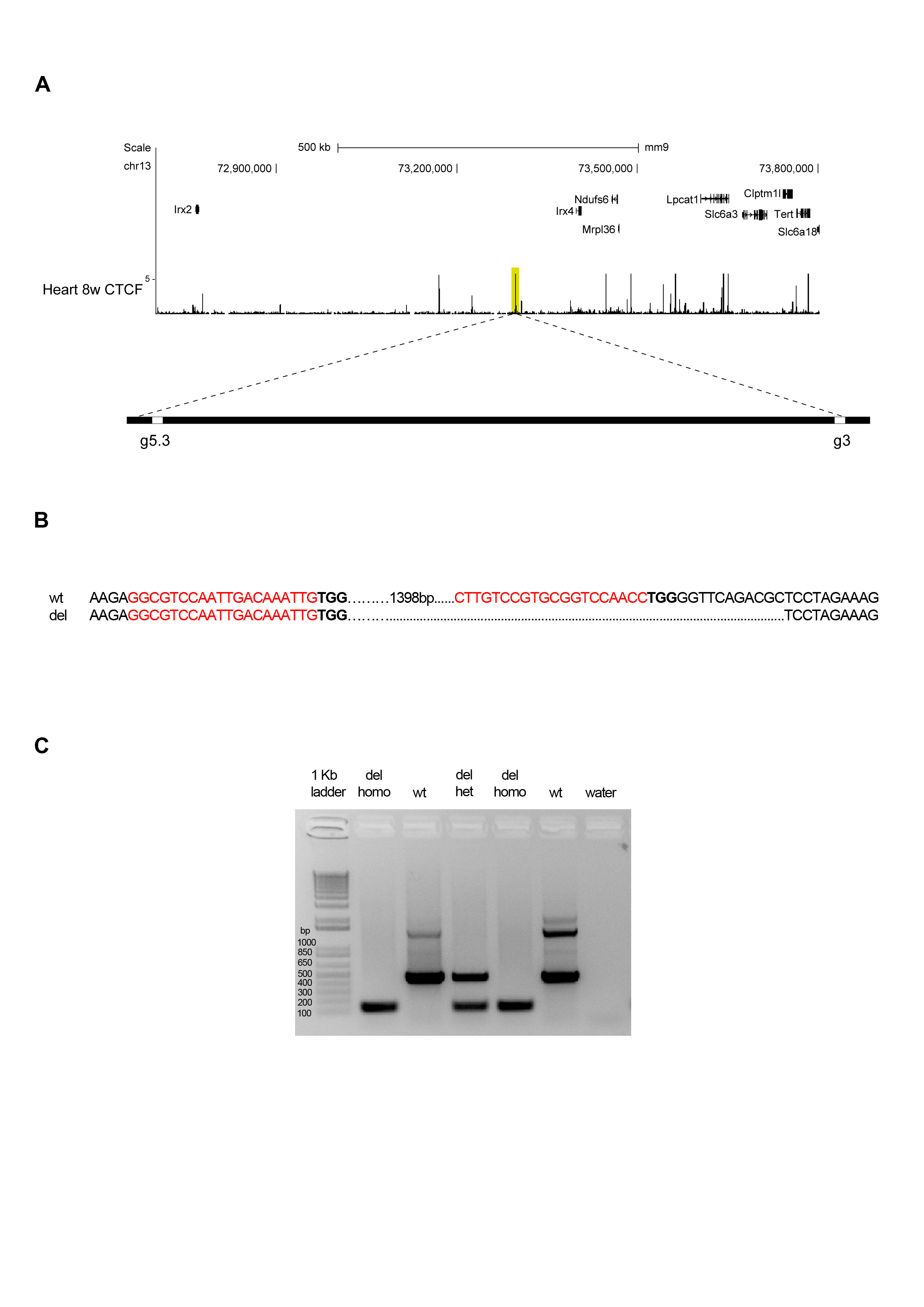

Supplement: S8 Fig — (A) Top, ENCODE data for CTCF binding in 8 weeks mouse heart (mm9 chr13:72700492–73802866). The Irx2/Irx4 intergenic CTCF BS is highlighted in green. Bottom, schematic representation of the location of the guide-RNAs used for genome editing of the CTCF BS. (B) Sequence of the wild type (wt) and deleted (del) alleles of the CTCF BS deleted. In red are the gRNAs sequences are indicate in red, and PAM sequences in bold lettering. (C) PCR genotyping of the different genotypes from the CTCF BS deleted mouse line. (TIF) [file pgen.1006985.s008.tif]
