# Supplementary material for: CTCF counter-regulates cardiomyocyte development and maturation programs in the embryonic heart
Source: PLoS Genet. 2017 Aug 28;13(8):e1006985. doi: 10.1371/journal.pgen.1006985 (PMC5591014; doi:10.1371/journal.pgen.1006985)
Supplement: S1 Table — (PDF) [file pgen.1006985.s009.pdf]

S1 Table. Genotypes of live embryos obtained from ♂*Ctcf*$^{fl/+}$;*Nkx2.5-cre*$^{tg/+}$ X ♀*Ctcf*$^{fl/fl}$ crosses

**stage**	**genotype***				**total**
	***Ctcf*$^{fl/+}$**	***Ctcf*$^{fl/fl}$**	***Ctcf*$^{fl/+}$; *Nkx2.5-cre*$^{tg/+}$**	***Ctcf*$^{fl/fl}$; *Nkx2.5-cre*$^{tg/+}$**	
E8.5	2	3	1	2	8
E9.5	26	29	27	26	108#
E10.5	58	58	59	46	221#
E11.5	137	150	132	128	547§
E12.5	15	20	12	14	61
E13.5	8	9	3	0	20
E14.5	7	6	3	0	16
E15.5	4	2	1	0	7

* equal numbers of embryos from each genotype are expected from this cross

# includes embryos used for in situ hybridization

§ includes embryos used for 4C-seq
