# Supplementary material for: CTCF counter-regulates cardiomyocyte development and maturation programs in the embryonic heart
Source: PLoS Genet. 2017 Aug 28;13(8):e1006985. doi: 10.1371/journal.pgen.1006985 (PMC5591014; doi:10.1371/journal.pgen.1006985)
Supplement: S2 Table — (PDF) [file pgen.1006985.s010.pdf]

S2 Table. Quantification of double positive CT3-CTCF nuclei in E9.5 mutant hearts

Ctcf $^{fl/fl}$***;Nkx2.5-Cre*** **E9.5**		
**sample***	**nuclei**	**CTCF + #**
1	684	302
1	661	455
1	635	349
1	630	396
1	593	171
2	550	245
2	559	283
2	560	433
2	538	133
2	427	217
3	515	111
3	515	116
3	558	271
3	523	199
3	505	238
total	***8453***	***3919***

* sample refers to independent embryos from which 5 sections were quantified

# only CT3 positive nuclei were scored for CTCF

(in control embryos, all CT3 positive cells are also CTCF positive)
