# Supplementary material for: CTCF counter-regulates cardiomyocyte development and maturation programs in the embryonic heart
Source: PLoS Genet. 2017 Aug 28;13(8):e1006985. doi: 10.1371/journal.pgen.1006985 (PMC5591014; doi:10.1371/journal.pgen.1006985)
Supplement: S3 Table — (PDF) [file pgen.1006985.s011.pdf]

S3 Table. Quantification of proliferation and apoptosis in E10.5-E11.5 control and mutant hearts

**control E10.5**			
**sample***	**CT3 + nuclei**	**PH3 +**	**TUNEL+**
1	785	6	0
1	894	28	0
1	618	82	0
1	588	48	0
1	650	8	0
2	866	12	0
2	997	14	0
2	1007	34	0
2	930	192	1
2	689	16	0
3	897	31	0
3	954	9	0
3	842	11	0
3	819	89	1
3	878	91	0

***Ctcf* $^{fl/fl}$*;Nkx2.5-Cre* E10.5**			
**sample***	**CT3 + nuclei**	**PH3 +**	**TUNEL+**
1	1081	11	0
1	745	11	0
1	837	142	1
1	735	27	0
1	777	16	0
2	1205	19	0
2	1091	28	0
2	1101	13	0
2	1216	44	0
2	1154	160	0
3	1284	30	0
3	1533	19	3
3	1466	19	3
3	728	17	1
3	910	21	1

**control E11.5**			
**sample***	**CT3 + nuclei**	**PH3 +**	**TUNEL+**
1	1676	287	0
1	1841	119	1
1	918	102	0
1	738	110	0
1	1636	251	0
2	2376	270	5
2	1759	175	2
2	1425	129	2
2	1658	271	2
2	1810	264	1
3	2034	108	4
3	2039	130	0
3	1958	268	0

***Ctcf* $^{fl/fl}$*;Nkx2.5-Cre* E11.5**			
**sample***	**CT3 + nuclei**	**PH3 +**	**TUNEL+**
1	1883	85	6
1	2021	180	3
1	1391	282	2
1	2083	279	0
1	2014	397	6
2	1810	260	1
2	2028	101	1
2	1637	114	3
2	1399	249	4
2	1696	151	2
3	1787	111	2
3	1539	139	0
3	1475	84	1

* sample refers to independent embryos from which 3-5 sections were quantified
