# Supplementary material for: CTCF counter-regulates cardiomyocyte development and maturation programs in the embryonic heart
Source: PLoS Genet. 2017 Aug 28;13(8):e1006985. doi: 10.1371/journal.pgen.1006985 (PMC5591014; doi:10.1371/journal.pgen.1006985)
Supplement: S7 Table — (PDF) [file pgen.1006985.s015.pdf]

S7 Table. Motif discovery in proximal and distal CTCF peaks of dysregulated genes in *Ctcf*$^{fl/fl}$;*Nkx2.5-cre*$^{tg/+}$ E10.5 hearts

		***de novo* motifs**	**p-val**	**known motifs**	**adj p-val**
**development**	**proximal**	*none*		CTCF	0,001
	**distal**	BORIS*	1,00E-11	CTCF / BORIS*	0 / 0
**translation**	**proximal**	*none*		CTCF / BORIS*	0 / 0
	**distal**	RPN4**	1,00E-13	CTCF / BORIS*	0 / 0,0001
**mitochondria**	**proximal**	BORIS	1,00E-15	CTCF / BORIS*	0 / 0
	**distal**	*none*		CTCF	0,0001

* CTCF-like factor

** Yeast C2H2 zinc-finger factor; second best match to the *de novo* motif is CTCF
