# Supplementary material for: CTCF counter-regulates cardiomyocyte development and maturation programs in the embryonic heart
Source: PLoS Genet. 2017 Aug 28;13(8):e1006985. doi: 10.1371/journal.pgen.1006985 (PMC5591014; doi:10.1371/journal.pgen.1006985)
Supplement: S8 Table — (PDF) [file pgen.1006985.s016.pdf]

S8 Table. Primers and oligonucleotides used in this study

Genotyping primers		
**Primer**	**Sequence**	**Notes**
lacZ_F	GCGACTTCCAGTTCAACATC	genotyping of transgenic embryos
lacZ_R	GATGAGTTTGGACAAACCAC	genotyping of transgenic embryos
Myogenin_F	CCAAGTTGGTGTCAAAAGCC	control for transgenic genotyping
Myogenin_R	CTCTCTGCTTTAAGGAGTCAG	control for transgenic genotyping
NKX2.5-Cre	GATGACTCTGGTCAGAGATACCTG	Forward for *Nkx2.5-Cre* allele
NKX-S	GCCCTGTCCCTCAGATTTCACACC	Forward for *Nkx2.5* wildtype allele
Nkx-AS	GCGCACTCACTTTAATGGGAAGAG	Reverse common primer for *Nkx2.5*
p8563	CTAGGAGTGTAGTTCAGTGAGGCC	Forward common primer for *Ctcf*
p8946	GCTCTAAAGAAGGTTGTGAGTTC	Reverse for *Ctcf* wildtype allele
p261	CGGCATCAGAGCAGCCGATTG	Reverse for *Ctcf* floxed allele
p260	TGTCACTAATCTCCACCTCACAG	Reverse for *Ctcf* deleted allele
CTCFdel_F	AGGCCTTTAGCAGGAACCTC	genotyping of CRISPR/Cas9 deletion
CTCFdel_R	CGATCCTGGAACAGGAAAAA	genotyping of CRISPR/Cas9 deletion

* F stands for forward primer and R stands for reverse primer

Primers for in situ hybridization probe generation	
**Primer**	**Sequence**
*Tnni2*_F	ACGTGGCTGAAGAGGAGAAA
*Tnni2*_R	TATTGGAGCGAGGCCAAGTA
*Tnni3*_F	GAGACCTCCAAGGTCACCAG
*Tnni3*_R	TTAAACTTGCCACGGAGGTC
*Tnnt1*_F	TGCACTAAAAGACCGCATTG
*Tnnt1*_R	TGGGGGCACTTTATTTTGAG
*Tnnt3*_F	GAAACCAAGACCCAAACTTAC
*Tnnt3*_R	TTTATTCCTAGACCCCAGAAG
*Ndufs6*_F	GGTTTCGGGGTTCAAGTGT
*Ndufs6*_R	TGGTGGGAGCATCCTTTATT
*Hopx*_F	AGCAGACGCAGAAATGGTTT
*Hopx*_R	CCCCTGCCTGTTCTGTTATC
SP6 promoter	ATTTAGGTGACACTATAGAA
T7 promoter	GTAATACGACTCACTATAGGG

* F stands for forward primer and R stands for reverse primer

4C viewpoint primers	
**Primer**	**Sequence**
*Irx4*_DpnII	TCCGGCGCAAGAGCGATC
*Irx4*_Csp6	GTAAGCGGATGGGAAGGAC
*Ndufs6*_DpnII	AGCATGTGTTGTTTGGGATC
*Ndufs6*_Csp6	AACACTGCGCACAGTTAAGC
CTCF BS_DpnII	TGGAACCCACAGTGCTGATC
CTCF BS_Csp6	ACCAGCAAATTAATCTACAAGGC
Adaptor reading primer	AATGATACGGCGACCACCGAACACTCTTTCCCTACACGACGCTCTTCCGATCT
Adaptor non reading	CAAGCAGAAGACGGCATACGA

*The reading primers have DpnII and non reading primers have Csp6

Oligos for Guide RNAs		
**Oligos**	**Sequence**	**Location**
g5.3	GGCGTCCAATTGACAAATTG / PAM: TGG	chr13:73297211-73297230
g3	CTTGTCCGTGCGGTCCAAC / PAM: TGG	chr13:73298566-73298585
